# Progressing Plastics Circularity: A Review of Mechano-Biocatalytic Approaches for Waste Plastic (Re)valorization

**DOI:** 10.3389/fbioe.2021.696040

**Published:** 2021-06-22

**Authors:** Efstratios Nikolaivits, Brana Pantelic, Muhammad Azeem, George Taxeidis, Ramesh Babu, Evangelos Topakas, Margaret Brennan Fournet, Jasmina Nikodinovic-Runic

**Affiliations:** ^1^Industrial Biotechnology & Biocatalysis Group, Biotechnology Laboratory, School of Chemical Engineering, National Technical University of Athens, Athens, Greece; ^2^Eco-Biotechnology & Drug Development Group, Laboratory for Microbial Molecular Genetics and Ecology, Institute of Molecular Genetics and Genetic Engineering, University of Belgrade, Belgrade, Serbia; ^3^Athlone Institute of Technology, Athlone, Ireland; ^4^AMBER Centre, CRANN Institute, School of Chemistry, Trinity College Dublin, Dublin, Ireland

**Keywords:** plastic waste, pretreatment, biodegradation, valorization, upcycling, depolymerase

## Abstract

Inspirational concepts, and the transfer of analogs from natural biology to science and engineering, has produced many excellent technologies to date, spanning vaccines to modern architectural feats. This review highlights that answers to the pressing global petroleum-based plastic waste challenges, can be found within the mechanics and mechanisms natural ecosystems. Here, a suite of technological and engineering approaches, which can be implemented to operate in tandem with nature’s prescription for regenerative material circularity, is presented as a route to plastics sustainability. A number of mechanical/green chemical (pre)treatment methodologies, which simulate natural weathering and arthropodal dismantling activities are reviewed, including: mechanical milling, reactive extrusion, ultrasonic-, UV- and degradation using supercritical CO_2_. Akin to natural mechanical degradation, the purpose of the pretreatments is to render the plastic materials more amenable to microbial and biocatalytic activities, to yield effective depolymerization and (re)valorization. While biotechnological based degradation and depolymerization of both recalcitrant and bioplastics are at a relatively early stage of development, the potential for acceleration and expedition of valuable output monomers and oligomers yields is considerable. To date a limited number of independent mechano-green chemical approaches and a considerable and growing number of standalone enzymatic and microbial degradation studies have been reported. A convergent strategy, one which forges mechano-green chemical treatments together with the enzymatic and microbial actions, is largely lacking at this time. An overview of the reported microbial and enzymatic degradations of petroleum-based synthetic polymer plastics, specifically: low-density polyethylene (LDPE), high-density polyethylene (HDPE), polystyrene (PS), polyethylene terephthalate (PET), polyurethanes (PU) and polycaprolactone (PCL) and selected prevalent bio-based or bio-polymers [polylactic acid (PLA), polyhydroxyalkanoates (PHAs) and polybutylene succinate (PBS)], is detailed. The harvesting of depolymerization products to produce new materials and higher-value products is also a key endeavor in effectively completing the circle for plastics. Our challenge is now to effectively combine and conjugate the requisite cross disciplinary approaches and progress the essential science and engineering technologies to categorically complete the life-cycle for plastics.

## Graphical Abstract

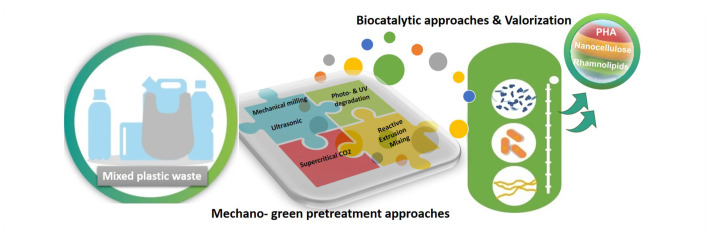

## Introduction

The prosperity of planet Earth is recognized as the foundation for the wellbeing of its populations. Global resource consumption rates are currently increasing at rates that would require the equivalent of almost three planets to sustain current lifestyles by 2050. Resource extraction and processing produce almost half of current greenhouse gas emissions and are responsible for more than 90% biodiversity loss and water stress. This coincides with predictions that global waste production is to increase by 70%. Plastic, which is primarily processed from fossil fuel resources, is a ubiquitous and indispensable material in the world economy and our daily lives, providing both high performance energy saving benefits along with alarming pollution and waste stockpiles. The predominant consumer petroleum-based synthetic polymers [low-density polyethylene (LDPE), high-density polyethylene (HDPE), polyvinyl chloride (PVC), polystyrene (PS) and polypropylene (PP), polyethylene terephthalate (PET) and polyurethanes (PU)], take hundreds of years to degrade in the environment, ensuring a long-lasting blight on our oceans, countryside and recently acknowledged to be dispersed through the food chain. As the production of plastic is expected to double over the next 20 years, plans such as legislation requiring all plastics packaging within the EU market to be either reusable or recyclable in a cost-effective manner by 2030 are increasingly important. It is now widely recognized that changing from consumption and transitioning to sustainable growth models is essential to safeguarding the planet and people. The development of new regenerative technologies is essential to eliminating the indelible imprint of pervasive plastic, to deliver plastics circularity and secure the future prosperity of the planet and its inhabitants.

In the case of all of these polymers, the steps to complete the lifecycle and the management of post-consumer plastics has not yet been adequately developed or implemented. The unabated demand for plastic products, the absence of appropriate post-use recycling and the ubiquitous environmental pollution command a paradigm shift in plastics technologies to meet this prodigious global challenge. A switch from outdated linear resource extraction, use and dispose model to a fundamentally circular modality is imperative. Post use and end of life plastics need to be revalorized as new products in a continuous use modality instead of disposal as waste. Pioneering technologies and innovative scientific developments are essential in the mission (Graphical Abstract) to transition from the linear to the circular plastic economy. Inspiration on the routes to pursue is available from nature, which readily operates elegant and efficient regenerative cycles for natural polymers and other waste streams (Vincent et al., 2006). Nature’s biodegradation and bioregeneration processes combine environmental weathering, microbial and enzymatic biocatalytic activities for depolymerization of post use plastics into constituent building blocks. Biochemical synthesis and repolymerization has the potential to revalorize these building-block molecules as functional bio constructs to complete the loop to enable continuous life cycle operation for the next generation of plastic materials and products. Combination and convergence of mechanisms spanning the disciplines of mechanical engineering, green chemical science, biocatalysis and bioprocessing is imperative to the completion of the life-cycle for plastics.

### Biomimetic Approaches to Plastic Circularity

While nature is expected to independently achieve the degradation and regeneration of waste PET, PE, PS and PU plastics over the course of the next centuries and millennia, it is recognized that, given the current pollution rates, this timescale will not be sufficient to protect the wellbeing of our planet. Modern science and engineering are well equipped to enhance and adopt biomimetic approaches guided by the myriad of natural regenerative cycles within nature. This report presents a review of mechanical and biocatalytic approaches as critical central technologies to the delivery of accentuated equivalent processes to accelerate plastics degradation and generate/regenerate plastic life cycles. The framework for a combined mechanical and biocatalytic approach is based on identifying that each of nature’s steps in a given regenerative cycle can be substituted with equivalent, yet augmented bio mimic ecological processes. In the case of naturally occurring biopolymeric materials, recycling can typically include: (1) environmental weathering and arthropodal excavation of a given biomaterial, (2) microbial and enzymatic digestion and building block generation, (3) biochemical re-assembly of building blocks and (4) biosynthesis of new biopolymers or biomaterials and incorporation within a bio-organism or bio-entity. Mechanical, irradiative and green chemical pretreatments can be used to simulate environmental weathering and arthropodal actions occurring in step 1. Comparable to natural or improved microbial and enzymatic digestion occurring in step 2 can be achieved using newly discovered strains, directed evolution of enzymes and novel microbial consortia development for high yield monomer and oligomer building block production from waste plastics. For step 3, current bioplastic producing strains, newly discovered and genetically modified strains under optimized conditions can be used to bioprocess these monomer and oligomer feedstocks to biopolymers with chemical structures agreeable to high performance bioplastics. Step 4 will require new development in bioplastic processing involving formulation, compatibilization, blending and compounding to produce new high performing bioplastics, comparable and equivalent to current market leading petroleum-based plastics, fully encompassing biodegradability functionalities. The proficient development of accentuated equivalent natural routes for waste plastic circularization promises a fully resource neutral, low carbon and energy process, which has the potential to remove the recalcitrant nature of the mainstay petroleum-based plastics, facilitate a seamless transition to new sustainable bioplastics and spur the development of a whole array of new bio-based technologies and materials promoting a new wave of bio-economic activities.

The quest to delivering accentuated natural bio routes for mainstay petroleum-based waste plastic, commences with addressing their inherent incompatibility with biological systems. Microbial biodegradation of polymeric materials can be divided into 4 stages: biodeterioration, biofragmentation, assimilation and mineralization (Ru et al., 2020; Samak et al., 2020). Biofragmentation includes the production of extracellular enzymes, stage that has not been widely studied. These produced enzymes can then break down the long polymer chains into monomers and oligomers, compounds that can penetrate the cell membrane more easily. As strongly bio-inert materials, petroleum-based plastics present a series of inhibiting factors, impeding the bioprocessing of these polymers. Low specific surface area, smooth surface topographies, high crystallinity, lack of accessible carbonyl groups and other hydrolyzable chemistries with extensive hydrophobic units, render these polymers insoluble in water, restrict microorganism attachment and assimilation, limit enzymatic adsorption and biodegradative catalytic activities. The initial approaches used pyrolysis or gasification of waste plastics to produce biofuels and syngas as a microbial feedstock (Kenny et al., 2008; Guzik et al., 2014). Low yield production of polyhydroxyalkanoates/polyhydroxybutyrate (PHA/B) biopolymers has been achieved by the microbial fermentation of syngas, however, the technology has proven challenging. Biodigestion studies of plastics are revealing microorganisms that can degrade plastics to varying degrees under specific conditions and over extensive timeframes. An important issue that occurs in microbial digestion is the resource loss, especially when mineralization occurs where the plastic constituents are reduced to CO_2_ and water. On the other hand, plastic depolymerization into its monomer and oligomer components can be achieved to limited degrees using enzymes derived from microorganisms. Time scales, quantities of plastics degraded and the lack of capacity to treat mainstay high crystallinity regions are currently limiting factors. Both bio-discovery and bioengineering for improved enzymatic degradation efficiencies of post-consumer plastic is now under intense development. In contrast, currently there are relatively few reports on degraded plastic constituent molecular building block processing or repolymerization either synthetically or using biotechnologies for the generation of new polymers or bioproducts. The capacity to biodigest and biodegrade waste plastics and convert the building blocks into biopolymers by ecologically agreeable processes, such as bacterial fermentation and enzymatic polymerization, provides means for unlocking the recalcitrant nature of plastics and eliminating the impact of the plastic pollution blight.

### The Recalcitrant Plastics

Plastics are in many aspects a remarkable material and have been instrumental in propelling much of recent rapid human progress. The inventors of plastic polymers in the early to mid-20th century, could not have foreseen the full impact, both positive and negative of future plastic products. Human manipulation of hydrocarbons has facilitated much of our social, technical and economic advancement. In the past 75 years the growth of plastics production has substantially outpaced any other manufactured material. Polymers such as PE, PS, PET, and PU all have simple molecular structures comprising chemically defined macromolecules. A combination of chemical and process engineering over the past decades has keenly honed the structural level features of these polymers to achieve associated high mechanical property performance. Good mechanical and fluid barrier properties within lightweight materials has spurred a fast-paced socio-economic development. Petroleum-based plastics, such as PET and PE, achieve the required degrees of high mechanical strength combined with flexibility and strong liquid and gas barrier properties by packing their sleek chemically structured chains into signature crystalline and amorphous regional arrangements. These same features, however, also prohibit degradability, in particular biodegradability. The tight alignment of chemically simple chains at high degrees of crystallinity renders these plastics largely incompatible with enzymatic hydrolysis and bioactivity. PE, PS, and PET are known as thermoplastics, since they are softened or melted on heating and then shaped, formed, welded, and solidified when cooled. Multiple cycles of heating and cooling can be repeated, allowing reprocessing and recycling. In addition, PU is also a resilient, flexible, durable and affordable manufactured material used in a very broad range of products.

Polyethylene (PE) is considered to be the most commercially produced plastic. Its annual production in 2016 exceeded 100 million tons, occupying a share of 30% in total plastic production globally (Plastics Europe, 2018). PE is a polymer of ethylene with a melting point in the range of 105–140°C (Omnexus Specialchem, 2020) and is easy to fabricate at low cost. It was first produced at an industrial level early in the 20th century. The most common PE forms in use are LDPE, HDPE and linear low-density polyethylene (LLDPE), delivering multiple malleable properties such as durability, chemical and abrasion resistance, pliability, strength (impact resistance) light-weighting and ease of weld. The different forms of PE can be used for products ranging from orthotics and prosthetics, fibers, textiles, pipes, marine constructs, as well as for plastic bags, food packaging and containers. However, notwithstanding its considerable advantageous properties, PE is one of the most recalcitrant pollutants, with very limited recycling implemented to date and is responsible for over 20% of the plastic packaging waste ended up in landfills.

Polyethylene terephthalate is a thermoplastic polymer with excellent physical and chemical properties. The global production of PET will be approximately 74 million tones by the end of 2020, with the largest part of its production to be used for water bottles and food or drink packages (Danso et al., 2018). Specifically, more than 480 billion bottles were produced worldwide in 2015, a number that is predicted to exceed 580 billion by 2021 (Choudhary et al., 2019). Despite the fact that PET is a fully recyclable polymer, less than 28% of PET bottles were recycled in the United States in 2018, while 57% of the produced bottles were disposed in landfills (Statista, 2018).

PS is widely utilized particularly in packaging, medicine and electronics. World PS production accounts for almost 4% of the total plastic production with annual production in 2016 of approximately 14.7 million tons (PlasticsInsight, 2020). PS is considered to be an inexpensive, heat resistant, light weighted and tough material and is mainly used in the form of expanded polystyrene (EPS), extruded polystyrene (XPS) -or Styrofoam- and high impact polystyrene (HIPS) (PlasticsInsight, 2020). The degradation of PS wastes can take place using various physicochemical methods, such as photo-oxidation, oxidation with peroxides and trace metals and chemical decomposition. Even though these methods can decompose polystyrene up to 70–90% (Savoldelli et al., 2017), they often use chemical reagents which can in turn have negative environmental impacts, particularly when implemented for larger-scale processes.

PUs make up 7.7% of the global plastic production (Gadhave et al., 2019), accounting for about 18 million tons a year (Wierckx et al., 2018). Despite a common name, PUs are a very heterogeneous group of compounds obtained by the polyaddition reaction of polyisocyanates and polyols. By utilizing different polyisocyanates and different polyols, a huge number of chemically and physically diverse PU can be produced and depending on the polyol used, the two main types are polyester PU and polyether PU (Howard, 2012). The degradation response of PU depends on the chemical format. Polyester PU has increased degradability compared to that of polyether PU, with aliphatic isocyanate based PU exhibiting distinctive degradation compared to aromatic isocyanate based PU (Mahajan and Gupta, 2015). Due to their highly divergent structures, when investigating the biodegradation of PU, attention to the specific composition is important as this directly correlates with the biodegradation response.

### The BioPlastics

Bio-based plastics and biopolymers are a growing economic sector, yet only accounting for circa 1% of the total plastic production. Price competitivity, decreased mechanical performance compared to fossil fuel-based plastics and incompatibility with established recycling infrastructures are limiting factors in the current market uptake of biodegradable plastics. Biodegradable and bio-based polyesters, such as polylactic acid (PLA), PHA/B and polybutylene succinate (PBS) and even petro-based polycaprolactone (PCL), have great potential for development to fulfill these requirements and exhibit advanced properties (mechanical, gas barrier performance and good processability) which provide prospective alternatives to their fossil-based counterparts, while fulfilling circularity and sustainability criteria (Jeremic et al., 2020). Polyester bio-plastics are currently expected to constitute the main drivers of the bio-based plastic market in the upcoming years, in particular for the largest market segment which is food and drink packaging.

Polylactic acid is a bio-based aliphatic polyester, produced from renewable resources such as starch and cellulose (Pranamuda et al., 1997). The three stereoisomers of PLA are poly(L-lactide) (L-PLA), poly(D-lactide) (D-PLA) and poly(DL-lactide) (DL-PLA) (Tokiwa et al., 2009). Since its commercial production in the late 1990’s PLA has replaced petrochemical-based polymers in certain food packaging, electronics and synthetic fiber applications (Karamanlioglu et al., 2017). However, extensive use has resulted in large amounts of PLA plastic ending up in landfills and contributing to environmental pollution. PLA is often very stable in soil, particularly when modified with nucleating agents used to increase its crystallinity and thereby its resistance to hydrolysis (Sun et al., 2019). Achieving PLA biodegradability, though less arduous than the case of fossil-based plastics, nevertheless needs to be addressed to establish its position as a fully sustainable biopolymer.

PHAs are microbial polyesters of various hydroxyalkanoates, starting with polyhydroxybutyrate (PHB). Polyhydroxyalkanoates (PHA/B) are synthesized as carbon and energy storage compounds during unbalanced cell growth. Due to the vast diversity of PHAs, this class of biopolymers can have diversified properties, tailored to a specific application. Today many companies produce PHA materials or products such as shopping bags, composting bags, household utensils, coating material of containers, razors and papers (Israni and Shivakumar, 2019). Additionally, PHAs have found many applications in the biomedical field (Grigore et al., 2019). The first PHA was discovered by French microbiologist M. Lemoigne in the mid-1920s and was poly(3-hydroxybutyrate) [P(3HB)] from *Bacillus megaterium*. Subsequently several aerobic and anaerobic bacterial strains accumulating P(3HB) were identified. In the1970s P(3HB) was described as an energy storage polymer (similar to glycogen and starch) and soon after that, other monomers than just 3HB were also identified as part of that storage (Grigore et al., 2019). Today more than 90 bacterial genera (over 300 microorganisms) have been described to produce PHAs being able to utilize about 150 different monomers (Hazer and Steinbüchel, 2007; Raza et al., 2018; Grigore et al., 2019). PHAs have been structurally classified to three categories depending on the chain-length of their monomers: short (scl), medium (mcl), and long chain-length (lcl) PHAs, incorporating C3-C5, C6-C14, and >C14 acids, respectively. Additionally, PHAs can be homo- or co-polyesters with different monomer ratios (Israni and Shivakumar, 2019).

Polybutylene succinate is a highly crystalline, aliphatic polyester composed of succinic acid and 1,4 butanediol, which can be derived either from fossil or renewable sources (Xu and Guo, 2010). Traditionally, PBS polymer is formed via polycondensation, a process that demands high vacuum and the presence of organometallic or metal-oxide catalysts, such as titanium butoxide (Xu and Guo, 2010). However, a more environmentally friendly process has emerged through biocatalysis by using lipases, that have been found to catalyze polymerization under mild reaction conditions (Kobayashi et al., 2000). PBS has been commercially available since 1993 and due to its thermal and mechanical properties, it is mainly used in food packaging, bottles, bags, flushable hygiene products and mulch films (Xu and Guo, 2010; Gigli et al., 2016). In order to improve the properties of PBS, it is often copolymerized with other petroleum- or bio-based aliphatic polyesters changing its structure and morphology, thus affecting its (bio)degradation (Xu and Guo, 2010).

Polycaprolactone is an aliphatic polyester produced from fossil resources, first synthesized in the early 1930s (Woodruff and Hutmacher, 2010). PCL was originally applied in drug-delivery systems, but due to its slow degradation rate, it was replaced by other bio-based polymers such as PLA, which could release encapsulated drugs faster (Woodruff and Hutmacher, 2010; Mohamed and Yusoh, 2016). Recently, PCL and PCL-based PU have again attracted scientific attention, especially in the field of tissue engineering and regenerative medicine (Lee et al., 2018). Furthermore, the conversion of PCL to nanocomposites improves its properties and expands its uses in packaging sector, replacing other non-biodegradable materials (Khatiwala et al., 2008; Mohamed and Yusoh, 2016).

Poly(ethylene 2,5-furanoate) (PEF) is a very interesting potential alternative for PET. The mechanical performance of PEF is higher that of PET and has shown better enzymatic degradability in preliminary studies. Moreover, both the monomers that compose its chain, ethylene glycol and 2,5-furandicarboxylic acid can be derived from biomass, making it a sustainable bio-based polymer (Loos et al., 2020).

These increasingly sustainable bio-based plastics are expected to gradually substitute fossil-based plastics in the market place. In particular, PLA, PHB, PBS, and PCL have attracted much attention in recent years, as a replacement in a variety of applications for fossil-based products. However, under unmanaged conditions, these polymers can be similar to fossil-based plastics and create analogous environmental problems as fossil-based plastics (Jeremic et al., 2020; RameshKumar et al., 2020). Currently, the waste generated by biodegradable plastics is minimal, and their end-of-use management is becoming an issue with the wide adoption of these plastics in various commodity applications. It therefore becomes an imperative to develop the biomimetic approaches for bioplastics. The relatively reduced recalcitrance of bioplastics combined with the knowledge gained in developing mechano-biocatalytic based technologies for petroleum-based plastics circularity, would make the circular solutions for bioplastics readily achievable.

## Mechano-Thermo-Photo Irradiative and Green Chemical (Pre)Treatment of Plastics

**FIGURE 1:**
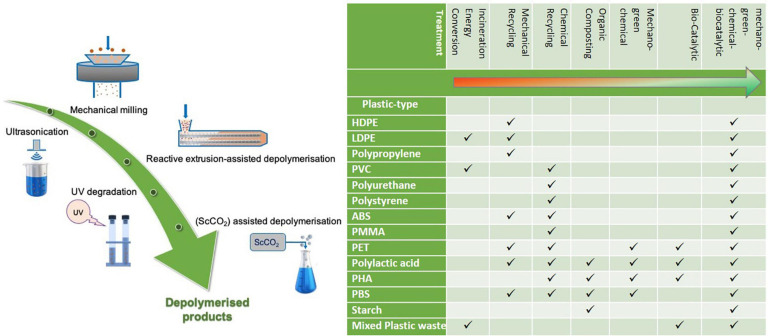
Mechanical, green chemical, and photo irradiation treatments for plastics which can be implemented to mimic and intensify natural weathering and arthropodal processing and comparative display of current mainstay post-consumer plastics management, alongside emerging mechano-green-chemical-bio-catalytic methodologies in terms of sustainability and circularity.

When polymers enter the environment, they undergo environmental weathering involving gradual natural degradation processes. Photodegradation, thermo-oxidative degradation and mechanical degradation are induced by the eroding effects of water, wind, UV irradiation and the geological habitat. This process is prolonged and depends upon the type of plastic and environmental conditions and can lead to the generation of microplastics (Andrady, 2011). Mechanical, green chemical and photo irradiation, such as those illustrated in Figure 1, can be used to mimic and intensify environmental weathering, providing a pre-treatment prerequisite for the degradation and valorization of plastic waste in subsequent biomimetic approaches. In order to ensure pretreatment processes are economically viable and environmentally safe, minimum energy requirements, restriction to green chemical reagents, and processes which are industrially scalable, are essential. Once an effective pretreatment has been carried out, the plastic waste can be rendered demonstrably more accessible for microbial and enzymatic degradation.

Weathering acts to biomechanically erode and fragment materials while UV induces changes on the molecular level. The combined effects act to increase porosity, decrease size, disrupt molecular chains, induce scissions and bond breakages, culminating in increased amenability to bio-catalytic processing and facilitating enzymatic adsorption. In the environment, plastic physical degradation and micro-plastic formation is induced by weathering, though not to the extent required to facilitate sufficient bio-assimilation and biofouling. Arthropodal activities involving boring, tunneling and feeding from biomaterials, as well as facilitation of microbial colonization, play a significant role in the efficient decay of vast quantities of biomass and nutrient repatriation. Intensified and tactical mechano-based treatments, which directly reflect and intensify environmental weathering can be implemented to initiate the deconstruction and ultimate (re)valorization of plastics. Physical, chemical and photo pretreatment methods can have a significant impact on polymeric structures, properties, composition, and reactivity, priming it for the subsequent steps in the process to accomplish plastic waste circularity.

Several studies have been reported the beneficial effects of various pretreatment methods for plastic waste to improve the accessibility to microbial degradation (Ru et al., 2020), with major pretreatments methods presented in the following sections.

### Mechanical Milling

Mechanical milling of plastic waste is an important pretreatment process for the reduction of plastic particle size and increase of its surface area. In the milling process, cutting elements of the rotor result in repeated fracturing, which can induce chain disorder and phase transformation, in addition to the primary aim to decrease particle size. A combination of shearing and fracture during the milling process can lead to chain scission and reduction in the molecular weight of plastics (Zhang, 2004; Tsai and Chen, 2017). The size and the properties of the milled particles depend on the type of the blades, rotation velocity, size of the input material, and process temperature. Generally the energy demand for milling process is high and energy efficiency of the milling and size reduction of plastics waste is currently under scrutiny (Marsh, 2001).

Pan and Shaw, explored mechanical milling as an approach for processing polymers to produce finer powders. It is observed that the milling performed at cryogenic temperature using liquid nitrogen found to be efficient in producing polymer composites (Pan and Shaw, 1995). Font et al., evaluated the amorphization of PET using the mechanical milling process and concluded that PET powder obtained is highly amorphous and can be recrystallized by heating the powders near to melting temperature (Font et al., 1997). The subsequent studies have focused on mechanical milling of various semi-crystalline polymers and their blends addressing the reduction in molecular weight, molecular weight distribution, change in morphology, free radical generation, and possible crosslinking products (Castricum et al., 1996; Rezaei et al., 2020). Smith at al. performed high-energy mechanical milling of poly(methyl methacrylate) (PMMA), polyisoprene (PI), poly(ethylene-alt-propylene) (PEP) at ambient or cryogenic temperatures (Smith et al., 2000). The authors concluded that substantial reduction in molecular weight and increased polydispersity occurs with an increase in milling time under ambient and cryogenic temperatures. Low energy wet grinding of PS, PLA, polycarbonate (PC), and PMMA found to be effective in reducing the particle size and altering the morphology of polymers (Ravishankar et al., 2018). The polymer powders produced under wet grinding conditions showed substantial chain scission, decreased molecular weight, and strains along the polymer backbone.

Milling methods, hence have potential as part of pretreatment process to progress the depolymerization potential of a range of petroleum-based polymers and for use in conjunction with other mechanical and green chemical methods to increase amenability to microbial and enzymatic activities.

### Ultrasonication

The application of ultrasonication for the implementation of polymer degradation is an emergent technology. Acoustic cavitation in which violently collapsing bubbles induce large shear fields can cause highly localized degradation of polymer chains. Exposure of polymeric macromolecules to high-energy ultrasonic acoustic waves can reduce molecular weight and induce chain scission, resulting in permanent viscosity reductions. This chain scission action and capacity to expose susceptible bonds is particularly important to new biocatalytic polymer degradation approaches. Increasing the accessibility of microbial enzymes to chemical group hydrolysis is essential in improving the amenability of synthetic plastics to biodegradation.

Ultrasonication induced degradation has been predominantly carried out on water soluble polymers usually from biomedical application field such as polyethylene glycol (PEG). A limited number of recalcitrant petroleum-based polymers have been subjected to ultrasonic disintegration with the effects of irradiation parameters (frequency, irradiation time, temperature, intensity, sonotrode depth) and the polymer characteristics (concentration, initial molecular weight, viscosity, polymer structure, alkyl groups) reported. Studies have included the role of additives including (air, radical scavenger, sodium chloride, titanium dioxide, and surfactants). A small number of investigations have been carried out to date on the combination of ultrasound and ultraviolet irradiation for enhanced polymer degradation. Quantification of the degradation achieved is typically carried out using change in intrinsic viscosity and molecular weight distribution analysis and the application of kinetic modeling. These models indicate different chain breakage mechanisms for ultrasonication and UV irradiation, while simultaneous exposure to UV and ultrasonic waves act to increase the number of scission products per breakage. A summary of the conditions and approaches applied are given in Table 1.

**TABLE 1:** Ultrasonic treatment conditions and induced degradation for selected petroleum-based plastics.

Polymer	Ultrasonic Degradation parameters	Degradation effects	References
HDPE	Irradiation time (0–600 s), Temperature (0–200°C), Power (0–300 watt)	Reduction in viscosity from 2.32 to 0.64 (l/g)	Li et al., 2005
LDPE	Irradiation time (0.3 s), Temperature (∼88°C), Frequency (22.5 kHz) Power (240 watt)	Reduction in viscosity from 0.06 to 0.052 (l/g)	Desai et al., 2008
PP	Irradiation time (0–200 min), Temperature (80–155°C), Frequency (25 kHz)	Degradation rate constant is decreased from 0.94 to 0.28 K x 10^13^ (mol^2^.lit^–2^s^–1^) as the temperature increased from 80 to 155°C	Chakraborty et al., 2004
PP	Combined with Melt apparatus, Irradiation time (0–5 min)	Reduction in dynamic Viscosity 800 to 40 (Pa.s)	Guo and Peng, 2007
PVP	Irradiation time (0–283 min), Temperature (∼21°C), Frequency (35 kHz) Power (80 watt)	Reduction in molecular weight 1.3 × 10^6^ to 1.5 × 10^5^ (g mol^–1^)	Akyüz et al., 2009
Polystyrene-polyacrylic acid	Combined with magnetic field, Irradiation time (6 h), Temperature (∼10–50°C), Frequency (35,53 kHz)	Reduction in molecular weight 2.5 × 10^5^ to 5.0 × 10^4^ (g mol^–1^)	Zhang et al., 2016

Polyethylene plastics have been demonstrated to undergo degradation upon ultrasonic irradiation. In HDPE reduced intrinsic viscosity was observed on increasing ultrasonic intensity (power) with reported molecular weight reduction leading to increased degradation (Li et al., 2005). The effect of different parameters on ultrasonic degradation of LDPE using viscometry showed that increase of reaction volume, reaction temperature and concentration, resulted in reduced extent of degradation (Desai et al., 2008). Variation of these parameters was associated with a decline in the cavitational phenomenon, which is a critical factor for induction of degradation.

Chakraborty et al. (2004) suggested that the degradation rate of polybutadiene and isotactic PP decreases by increasing temperature and vapor pressure of solvents. Both parameters tend to generate a cushioning effect during the cavitational phenomenon reducing degradation rate. Guo et al. revealed the chain scission behavior of PP melt under ultrasonic irradiation. An increase in crystallinity accompanied by a slight decrease in molecular weight was observed under these conditions. The degradation rate was noted to be relatively lower than solution-based ultrasonic degradation (Guo and Peng, 2007).

Using online light scattering measurements of the ultrasonic degradation rate of polyvinylpyrrolidone (PVP), the solvent (water/methanol) was observed to be a rate-determining parameter for polar polymers (Akyüz et al., 2009). Zhang et al. efficiently enhanced the degradation rate of the polystyrene-poly acrylic acid brush by coupling both magnetic field and ultrasonic irradiation. In the presence of the magnetic field, sonochemical efficiency increased 10-fold. Amplification of the magnetic field helped to decrease the viscosity of brush solution by boosting acoustic cavitation (Zhang et al., 2016).

### Photo- and UV Degradation

Photo- and UV induced polymer degradation has been widely demonstrated for an array of petroleum and biobased polymers. The extent and effectivity of the induced impact on molecular weight and hydrophobicity reduction varies with both polymer and conditions. Evaluation of the induced increased amenability of the plastics under photo or UV degradation treatment to bioactivity is lacking. An overview of the impacts of photo and UV treatments for the prevalent plastics is detailed here.

### Photodegradation

Commodity plastics do not contain chromophoric groups and the reactions that occur in the presence of sunlight are thermo-oxidative, photolytic and photooxidative in nature. Cross-linking reactions and chain scissions play a major role in the initiation of photodegradation. It is important to note that most of the commodity plastic contain stabilizers to protect from radiation. However, prooxidants and photocatalysts are used to enhance photooxidation. Previous studies proved that the combination of pro-oxidant and photocatalyst can significantly enhance photodegradation. Low dispersity of photocatalysts in polymer matrices currently restricts their large-scale use. A summary of photo-degradants and other process conditions reported are given in Table 2.

**TABLE 2:** Photodegradation parameters and induced degradation for selected petroleum-based plastics.

Polymer	Photo Degradation parameters	Degradation effects	References
LDPE	Wavelength (390 nm), Exposure time (45 days), Temperature (45°C)	Carbonyl index increase from 64 to 116	Gharehdashli et al., 2020
	Wavelength (280–320 nm), Exposure time (90 days), Temperature (45°C), Power of lamp (15 watt)	Arithmetic mean roughness increase from 2.7 to 71.8	Ranjan and Goel, 2019
	Wavelength (254–366nm), Exposure time (96 h), Power of lamp (20 watt)	Reduction in tensile strength up to 55%	Shawaphun et al., 2010
	Exposure time (up to 72 h), Temperature (23°C)	Carbonyl index increase of up to 65.58%	Ferreira et al., 2009
	Wavelength (254 nm), Exposure time (100 h), Power of lamp (30 watt), Temperature (25°C),	Surface modifications and new peaks were observed	(Ołdak et al., 2005)
	Exposure time 1200 h), Temperature (45°C)	Carbonyl index is increased from 0 to ∼0.9	(Eyenga et al., 2002)
PE	Wavelength (420 nm), Exposure time (38 days), Power of lamp (30 watt)	The reduction in weight-average molecular weight of up to 94.3%	(Fa et al., 2010)
PP	Wavelength (254 nm), Exposure time (100 h), Power of lamp (30 watt), Temperature (25°C)	Carbonyl index is increased from 0 to ∼0.4	(Eyenga et al., 2002)
PS	Exposure time (36 h), Power of lamp (400 watt), Temperature(25°C)	Chain scissions decreased the molecular weight, Weight loss up to −15%	(Nakatani et al., 2016)
	Exposure time (180 min), Power of lamp (4 watt),	Weight loss up to 18%	(Bandyopadhyay and Basak, 2007)

An increase in the crystallinity and carbonyl index was also observed after exposure of LDPE films to UV irradiations (Gharehdashli et al., 2020). Ranjan et al. indicated that the photodegradation of LDPE is faster in the air than its counterparts (double distilled water, two salt solutions with variable ionic strengths), under UV irradiation for 90 days. Generation of carbonyl and hydroxyl groups followed by surface roughness was observed, which helped to promote photo-oxidation. Oxygen showed favorable results in initiating photodegradation while salts impeded the initiation of photodegradation (Ranjan and Goel, 2019).

Polyethylene-TiO_2_ films were susceptible to increased photodegradation as compared to neat films by adding polyethylene (PE) wax which tends to increase the dispersity of TiO_2_ in polyethylene. Furthermore, the degradation behavior of LDPE and PP in the presence of different metal oxides (ZnO, TiO_2_, Fe_2_O_3_, CuO) under UV irradiation was also shown.

### UV-Degradation

Polymers subjected to UV radiation can rapidly undergo degradation process. The exposure of plastics to UV light will trigger photooxidation leading to the generation of carbonyl (-C = O) and vinyl (-CH_2_ = CH_2_) groups and chain scission of the polymer backbone. The degradation of polymer is associated with chain scission, reduction in molecular weight, crosslinking, secondary oxidation, and a significant diminish in the mechanical properties (McKeen, 2013). The presence of oxygen and moisture is significant for the initiation of degradation process and its sustenance, respectively.

UV irradiation and photochemical degradation of commercially available polymers PC, PP, PS, acrylonitrile butadiene styrene (ABS) and PMMA was investigated by Nagai et al. (Nagai et al., 2005). The studies showed that degradation exists near the surface region and shows extreme degradation due to the initial population of oxygen near the surface of the polymers. A comparative UV and thermo-oxidative degradation has been performed on commercial polymers such as PE, PP, polyamide PA6 and PBT (Gijsman et al., 1999). PE showed the lowest oxidative degradation among the polymers evaluated which is associated with the variation in oxygen diffusion within the different polymers. Hamad et al. assessed the photochemical degradation of PVA with continuous UV irradiation in the presence of hydrogen peroxide using UV photoreactor. Simultaneous exposure to UV light and continuous flow of H_2_O_2_ resulted in significant weight loss of polymer, reduction of molecular weight and total carbon content (Hamad et al., 2016). Films of PS were exposed to UV to mimic outdoor conditions (air, l^3^300 nm) and rapid yellowing and embrittlement was observed for PS films due to photooxidative degradation (Bottino et al., 2004). The mechanism of PS photolysis depends on the mobility of free radicals in the polymer matrix and their bimolecular recombination during UV exposure (Yousif and Haddad, 2013). The photodegradation of PS films was investigated in the presence of photocatalysts, such as benzophenone and thioxanthone (Pinto et al., 2013). The results suggested that photocatalysts can accelerate photodegradation and photo-oxidation processes in PS. Different photocatalytic systems evaluated for various commercial polymers are shown in Table 3.

**TABLE 3:** Photocatalytic degradation parameters and induced degradation for selected petroleum-based plastics.

Polymer	Photocatalytic degradation	Degradation effects	References
Polyethylene (LDPE)	TiO_2_ nanoparticles- 500 h of UV	Cavity formation, weight loss- 33% under visible light 60% under UV light after 90 days	Mehmood et al., 2016
	Polypyrrole- TiO_2_ nanocomposite	Release of volatiles, formation of cavities	Li et al., 2010
	ZnO–175 h under visible light	Increased brittleness withwrinkles, Formation of hydroperoxides, peroxides, carbonyl and unsaturated groups	Tofa et al., 2019
	TiO_2_ nanotubes	Increased crystallinity, improved carbonylindex 50% degradation under visible light in 45 days	Ali and Jamil, 2016
	TiO_2_-MWCNTs	Weight reduction- 35% in 180 h UV irradiation	An et al., 2014
	Copper phthalocyanine (CuPc) sensitized TiO_2_ photocatalyst	Chain scission reaction, reactive oxygen species (ROS) generation	Sökmen et al., 2017
Polypropylene	TiO_2_-rGO nanocomposite under sunlight (130 h)	Higher carbonyl index, appearance of cavity	Verma et al., 2017
Polystyrene	ZnO with photosensitizing dye under exposure to UV	Weight reduction – 16% Lower mechanical strength	Bandyopadhyay and Basak, 2007
Polyvinylborate	TiO_2_ nanoparticles under UV irradiation	Weight loss – 5–15%	Koysuren, 2018

### Supercritical Carbon Dioxide Assisted Depolymerization

Supercritical CO_2_ (ScCO_2_) is a versatile solvent and has a significant role in polymer processing and modification. The combination of gas like viscosity and liquid-like density of supercritical fluids makes them excellent solvents for various applications. The sorption of CO_2_ into polymers leads to swelling and results in changes in their physical, thermal, and mechanical properties (Kendall et al., 1999). The potential impact of scCO_2_ treatments for alteration of properties and features of plastics for increased amenability to biodegradation and microbial and enzymatic activities is also largely unexplored. This phenomenon, referred to as the plasticization of polymers, has a significant effect on the thermal and rheological properties of the polymer. The plasticization effect reduces the glass transition temperature of the polymer and provides access for CO_2_ molecules to interact with the functional groups and polymer backbone. Often scCO_2_ is used in the synthesis of polymers, polymer processing, creating polymer blends, increased diffusion of fillers, depolymerization, and modification of polymers owing to its attractive physical properties.

Over the last two decades, much attention has been paid to the use of scCO_2_ for processing and modification of various commercial fossil- and bio-based polymers (Yadav et al., 2020). Despite of increased use of scCO_2_ in polymer modification, only a limited number of studies are available on the use of scCO_2_ for polymer depolymerization. The majority of the studies to date focus on the use of scCO_2_ in the pyrolysis of plastic and other waste materials to increase the yield of hydrocarbons and production of nanomaterials (Feng and Meier, 2017; Montesantos and Maschietti, 2020). The depolymerization reaction proceeds effectively in the presence of scCO_2_ resulting in monomer production, which are recoverable with high yields (Goto, 2009). Depolymerization of PET was performed under pyrolysis conditions (at 500–650°C) in the presence of scCO_2_. It is noted that the presence of scCO_2_ favors the degradation of PET precursors into individual aromatic hydrocarbons more favorably (Wei et al., 2011). Under prolonged pyrosis conditions, the aromatic hydrocarbons were further decomposed to produce uniform, well-shaped, onion-shaped micro carbons and nanoflakes, which have potential applications as reinforcing fillers and conductive additives (Hu et al., 2014). Zhang et al. studied sustainable approach for disposal of PVC waste using scCO_2_. The maximum debromination was achieved in the presence of scCO_2_ while managing the degradation of PVC at optimum temperature, pressure, and time (Zhang and Zhang, 2020).

### Reactive Extrusion-Assisted Depolymerization

Reactive extrusion (REX) is most commonly used as a process in polymer compounding to modify the polymer properties and produce functionalized polymer composites (Fink, 2018). In a typical REX process, the extruder works as a continuous stirred reactor and it allows to carry out various modification processes in the absence of solvent to produce polymer composites in a single step. Several types of chemical reactions have been performed by reactive extrusion based on the incorporation of free radical initiators, functional groups, blending, coupling agents and can be summarized into the following six major categories (Brown and Orlando, 1988; Eisenbach and Heinemann, 1995; Narayan et al., 1998; Crawford, 2017) which are grafting, functionalization, controlled degradation, reactive blending, bulk polymerization and coupling reactions.

Several thermal pretreatment methods have been used for the degradation of polymers. However, most of the processes require a high energy input over long processing times and result in a lower degree of degradation. REX has been previously applied for controlled degradation of various polymers using free-radical initiators. REX is an efficient process and can be performed at lower temperatures, and high degradation rates can be achieved by choosing appropriate processing conditions and type of free-radical initiator (Kim and White, 1995; Fan and Feng, 2013). Pabedinskas et al. studied the degradation of PP in the presence of 2,5-dimethyl-2,5-bis(*t*-butylperoxy) hexane (DHBP), a dialkyl peroxide as a free-radical initiator using REX process (Pabedinskas et al., 1989). Both single screw and twin extruders were used for degradation studies, and levels of initiator seemed to have a significant role in the molecular weight distribution of degraded products. PP degradation, during multiple extrusion cycles at different temperatures, was studied by following chemical and molecular weight changes (González-González et al., 1998; Canevarolo, 2000). Three zones with different degradation-temperature behavior were monitored, and the increase in temperature did not have much effect on the number of chain scissions and molecular weight of degraded products. The catalytic degradation of HDPE was conducted in a single-screw extruder at reaction temperatures of 425°C, 450°C, and 475°C using silica-alumina as the cracking catalyst (Wallis et al., 2008). The degradation products obtained from REX process predominantly contained C5 products, and a kinetic model was proposed to compare the carbon number distribution of the reaction products.

Reactive extrusion depolymerization of PET was investigated in the presence of ethylene glycol using a single screw extruder (Mohsin et al., 2017). The degradation yield increased with increased (EG) content, and it was concluded that EG interacts with PET and acts as a plasticizer promoting the rate of degradation. REX of PET was carried out at high temperature and pressure using a co-rotating twin-screw extruder to study the hydrolytic depolymerization (Yalçinyuva et al., 2000). Process parameters such as screw speed, pressure and residence time were evaluated to achieve high depolymerization rates. It was observed that a high yield of low molecular weight products is obtained at low residence times, which indicates relatively high depolymerization rates under optimized process conditions. A single-step continuous REX-based process to depolymerize PET has been investigated (Patterson, 2007). The depolymerization is affected by the concentration of EG with the high molecular weight polymer (glycolysis). EG causes chain scission by attacking the ester linkages along the polymer backbone. The twin-screw extruder conveys PET and thus continuously creates fresh surface area that facilitates penetration of the depolymerizing agent into the polymer. Chen et al. (2016) recently reported UV-induced REX to control the chain scission of PLA. The addition of multifunctional chemical agent, trimethylolpropane triacrylate (TMPTA) promoted random main chain scissions of PLA under UV-induced reactive extrusion.

Reactive extrusion hence represents as a valuable pretreatment tool for petroleum and bio-based plastics. Studies and investigations can be performed to achieve tailored altered molecular weights and morphologies, chemical bond functionalizations and hydrophobicity reductions. In combination with other mechanical-green chemical treatments described, integrated, such as in the case of scCO_2_ or sequential such with UV or ultrasonication, pretreatment presents a powerful toolkit which has the potential for energy minimized, green chemical reagent assisted, rendering of a broad range of polymers for dramatic improved amenability to microbial and enzymatic depolymerization and ultimate (re)valorization.

On consideration of the most viable approaches which can mimic weathering and arthropodal activities, the issues of logistics, scalability, time constraints, energy consumption and economic costs are as important as technical efficiency. Furthermore, there is an evidential lack of studies correlating such pretreatment methodologies and biodegradation. Referring to recent results on combined ultrasonication and enzymatic treatment of demonstrating increased monomer output, subsequent enzymatic treatment post mechano-green chemical treatments is a highly promising route. As identification of the optimal mechano-green chemical treatment or sequence of treatments for each specific plastic and tailoring of these treatments to the chemical ad crystallinity structure of the individual polymers progresses, achievement of efficient degradation and depolymerization through combination mechano-green-chemical-biocatalytic treatments is a promising prospect (Figure 1).

As mechano-green-chemical and biocatalytic methodologies progress and present as industrially scalable options for post-consumer plastics management the advancement toward circularity is clear. A comparative display illustrates the potential impact of mechano-green-chemical and biocatalytic methodologies on sustainable circularity, alongside current mainstay approaches (Figure 1). These range from resource loss high carbon foot print options which include incineration and energy conversion. Mechanical recycling is an option which works for a limited number of cycles for plastics such as PET and hence presents as an interim carbon sink. Chemical recycling can incases be used to retrieve monomers for repolymerization, though solvent requirements and prolonged harsh conditions limit sustainability. Organic composting is feasible for biodegradable polymers, however this results in resource loss. Mechano-green-chemical methodologies have potential for the sustainable depolymerization of a number of polyester polymers. Bio-catalytic depolymerization currently has potential for the sustainable depolymerization of a limited number of plastics. Combined mechano-green-chemical-bio-catalytic methodologies clearly have potential to achieve full circularity for each of the plastics in a low carbon footprint and regenerative fashion.

## Microbial and Enzymatic Degradation of Plastics

Planet carbon cycle is a balance between photosynthesis and respiration that provides the required energy for sustaining life on earth through sunlight. Worldwide, a major fraction of terrestrial carbon is fixed in the form of plant derived lignocellulosic biomass. This plant cell wall material is made up of non-starch polysaccharides, such as cellulose and hemicellulose, as well as a non-carbohydrate aromatic heteropolymer named lignin. This complex wall protects plant cells from physical or biological damage, while assists plant growth by increasing the surface exposed to solar radiation for photosynthesis. Cellulose, the most abundant polysaccharide on earth, is a linear natural biopolymer consisting of glucose units linked by β-1,4-glycosidic bonds forming crystalline microfibrils via hydrogen bonding and van der Waals interactions (Horn et al., 2012). On the other hand, hemicelluloses are a heterogeneous group of polysaccharides consisted again of β-1,4-glycosidic bonds forming the backbone made of pentoses, hexoses and sugar acids, which are usually decorated with side chains (Saha, 2003). Last but not least, the main contributor in plant cell wall recalcitrance, is lignin, a non-carbohydrate polymer consisted mainly of phenylpropanoid units that are connected through free-radical oxidative polymerization of coniferyl, sinapyl and *p*-coumaryl alcohol derivatives (Vanholme et al., 2010). In addition to the main lignocellulosic components, cutin is a hydrophobic network that diminishes water loss constituting the outermost layer of the plants, which is composed of linear and branched oxygenated fatty acids and epoxide groups connected with ester linkages (Fich et al., 2016).

The degradation of this recalcitrant structure by microbial enzymes is a key process by phytopathogenic fungi and bacteria, essential for our planet’s carbon cycle. However, this plant cell wall, developed through millions of years of evolution, resulted in a strong recalcitrance network of biopolymers acting against pathogens, playing a major role as plant defense system. On the other hand, saprophytic microbes have evolved a great battery of enzymatic tools designed for the efficient degradation of the aforementioned components, secreting cocktails of different depolymerases (cellulases, hemicellulases, ligninases, as well as cutinases) that appear to be a very diverse set of enzymes, belonging to different families of glycosyl hydrolases, carbohydrate esterases and auxiliary activity enzymes that are members of different families of the Carbohydrate Active Enzymes (CAZy) database (Lombard et al., 2014). This powerful toolbox, has been evolved for the degradation of high molecular weight biopolymers that show crystallinity or hydrophobicity properties, similar to the manmade synthetic polymers.

Since plastic pollution on our planet is relatively recent compared to fungal and bacterial saprophyte evolution, corresponding plastic degrading enzymes are not expected to be evolved and potentially discovered through their presence in novel metabolic pathways capable of recycling carbon locked in manmade synthetic polymers contaminating our planet’s surface. Obviously, the expected biocatalytic treasure should be present in lignocellulosic secreted enzymes, especially the ones that are capable of degrading the non-carbohydrate components of plant cell walls, such as cutin and lignin other natural polymers such as rubber. As thoroughly discussed in the following paragraphs, there is a vast number of publications describing the synthetic polyester degradation of PET or PU, as well as biodegradable polymers such as PLA, PCL, PBS or PHA mediated by carbohydrate esterases, cutinases or cutinase-like enzymes and lipases, underpinning the significance of these enzymes, long known to the Industrial Biotechnology, in the field of polyester degradation (Table 4).

**TABLE 4:** Type of enzymes associated with the depolymerization activity of plastic materials.

Plastics	Type of enzyme
Poly(ethylene) (PE)	Laccase (EC 1.10.3.2), Manganese peroxidase (EC 1.11.1.13), Alkane monooxygenase (EC 1.14.15.3, 1.14.14.28)
Poly(ethylene terephthalate) (PET)	Cutinase (EC 3.1.1.74), Lipase (EC 3.1.13), Carboxylesterase (EC 3.1.1.1)
Poly(styrene) (PS)	Hydroquinone peroxidase (EC 1.11.1.7)
Polyurethanes (PU)	Urethanase (EC 3.5.1.75), Cutinase (EC 3.1.1.74), Esterase (EC 3.1.1.1), Aryl acylamidase (EC 3.5.1.13), Elastase (EC 3.4.21.36),
Poly(lactic acid) (PLA)	Lipase (EC 3.1.13), Cutinase (EC 3.1.1.74), Carboxylesterase (EC 3.1.1.1), Alkaline protease (EC 3.4.21.14)
Poly(hydroxyl- alkanoate) (PHB/PHA)	PHA/B depolymerases (EC 3.1.1.75 and EC 3.1.1.76), Lipase (EC 3.1.13)
Poly(butylene succinate) (PBS)	Lipase (EC 3.1.13), Cutinase (EC 3.1.1.74), Cholesterol esterase (EC 3.1.1.13)
Polycaprolactone (PCL)	Lipase (EC 3.1.13), Cutinase (EC 3.1.1.74)

**FIGURE 2:**
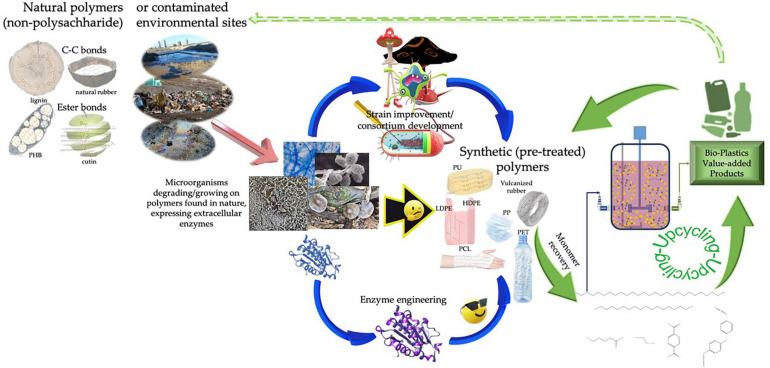
Overall approach to access new biocatalysts and to optimize them for the mixed plastic waste depolymerization and upcycling toward bio-plastics and other value-added compounds.

The more stable synthetic polymers consisted of strong C-C chemical bonds, such as PE, PP or PS, are resilient to biodegradation with oxidative enzymes, such as ligninases to be candidate targets for enzyme discovery toward the production of an efficient enzymatic cocktail capable of degrading these non-biodegradable synthetic polymers. As previously mentioned, and in similarity with lignocellulose enzymatic utilization, a pretreatment step is of utmost significance for the successful biodegradation of synthetic polymers. As a rule, materials with higher crystallinity are more resistant to enzymatic hydrolysis, therefore, biocatalytic efforts resort to protein engineering or the addition of greener additives from the selection of detergents, anionic/cationic surfactants, cofactors, hydrophobins, or others, techniques known to increase the enzymes’ adsorption on the polymer surface (Samak et al., 2020). The best performing enzymes associated with depolymerization activity of plastic substrates are highlighted in Table 5. Only recently pretreated post-consumer PET was successfully depolymerized into terephthalic acid and ethylene glycol at industrially relevant scale (16.7 g/L/h of terephthalate) using engineered PET depolymerize (Tournier et al., 2020). The quest for new microorganisms and enzymes is still very much alive and is twofold via traditional isolations and using multi-omics approaches (Jyotika et al., 2020). *Penicillium raperi*, *Aspergillus flavus*, *Penicillium glaucoroseum* and *Pseudomonas sp.* were isolated from soil, activated sludge, farm sludge, and worms’ excreta as the most capable plastic degrading microbes (Taghavi et al., 2021). Nevertheless, biotechnological degradation of majority of both recalcitrant and bioplastics is still at a very early stage of the learning and development curve. Therefore, this review also introduces EU Horizon 2020 research project BioICEP, “BioInnovation of a Circular Economy for Plastics” which concurrently with Mix-UP “Mixed plastics biodegradation and upcycling using microbial communities” project is addressing multiple aspects of the biological (microbial and enzymatic) depolymerization and valorization of the mixed plastic waste (Figure 2).

**TABLE 5:** The best performing enzymes associated with depolymerization activity of plastic substrates.

Plastics	The best performing enzyme	Material	Experimental conditions	Results	References
Poly(ethylene) (PE)	Alkane hydroxylase from *Pseudomonas aeruginosa* E7	LMWPE powder Mw 1.700	Compost, 37°C, 80 days 3.5 g LMWPE blended with 200 g (wet weight) sterilized compost. The mixture was inoculated with *Pseudomonas aeruginosa* E7	40.8% mineralization	Jeon and Kim, 2015
Poly(ethylene terephthalate) (PET)	TfCut2 from *Thermobifida. fusca* expressed in *Bacillus megaterium*	PET-CP, post-consumer Carton Pack (Carton Pack Srl, Rutigliano, Italy) Crystallinity 4–6%	70°C, 1.8 mL Phosphate pH 8, 7 d (17 mg film/mL reaction) 0.05 nmol enzyme/mg film or 1 nmol enzyme/cm^2^ film	23.9-56.6% weight loss	Wei et al., 2019
	Cut190* from *Saccharomonospora viridis*	PET-S from package 600 μm thick	63°C, 1 mL 0.1 M Tris pH 8.2, 50 mM CaCl_2_, 24% glycerol, 3 d (20–25 mg film/mL reaction) 11 nmol enzyme/mg film	27% weight loss	Kawai et al., 2014
Poly(styrene) (PS)	Hydroquinone Peroxidase from *Azotobacter beijerinckii* HM121	Dissolved PS (Aldrich Chem Co) Mn 930,000	0.4 ml of water, 10 mM hydrogen peroxide, 10 mM tetramethylhydroquinone, 100 mM potassium phosphate buffer pH 7.0, 30°C, 10 min 0.4 ml of dichloromethane containing 2 g/L of polystyrene. 2.4 U/mL of hydroquinone peroxidase (1.0 mg/mL protein)	*M*_*n*_ reduced to 350 and 1,000	Nakamiya et al., 1997
Polyurethanes (PU)	Lipase *Cryptococcus* MTCC 5455	Polyester PU (based on poly(diethylene glycol adipate) and 2,4 TDI)	PU cubes in buffer pH 6 Concentrated lipase (1500 U) 96 h at 30°C	96% weight loss with the production of diethylene glycol (DEG) and adipic acid (AA)	Thirunavukarasu et al., 2015
Poly(lactic acid) (PLA)	ABO2449 Esterase from *Alcanivorax borkumensis*	Solid PLA (Sigma-Aldrich) MW 1.0–1.8 × 10^4^	35°C, 1.0 ml 0.4 M Tris–HCl pH 8.0, 0.1% Plysurf A210G (detergent), 36 h 10–12 mg PLA powder/mL reaction 0.005 g enzyme/g PLA	Up to 90% conversion of PLA into lactic acid monomers and oligomers	Hajighasemi et al., 2016
Poly(hydroxyl- alkanoate) (PHB/PHA)	PHB depolymerase from *Paucimonas* (*Pseudomonas*) *lemoignei*	P(HB-*co*-10 mol%HV) Bacterial origin (Marlborough Biopolymers Ltd., England) *M*_*n*_ 96000 Chloroform-casted film *T*_*m*_ 145°C, *X*_*c*_ 61% 25 × 8x (0.2–0.3) mm	37°C, 1.5 mL 50 mM Tris–HCl pH 8, 1 mM CaCl_2_, 4 × 20 h 25 pmol enzyme/cm^2^ film/20 h	85% weight loss	Scandola et al., 1997
Poly(butylene succinate) (PBS)	Cutinase from *Fusarium solani*	PBS films Mn 15.0-21.0 × 10^4^ 30 mm × 10 mm 0.1 mm thick	40°C, 10 mL 20 mM Tris–HCl pH 8.0, 10 h 20 μg enzyme/mL reaction 0.33 μg enzyme/mm^2^ PBS or 0.014 nmol enzyme/mm^2^ PBS	100% weight loss	Hu et al., 2016
Polycaprolactone (PCL)	Cutinase from *Aspergillus fumigatus*	PCL film (mixing dichloromethane (20%) and ω-caprolactone monomer) 250 μm thick 1 cm^2^	40°C, 2,6 mL Tris pH 8.0, 6 h 0.011 g PCL/mL reaction 0.012 g enzyme/g PCL 0.002 mg enzyme/mm^2^ PCL	100% weight loss	Ping et al., 2017

### Microbial and Enzymatic Degradation of PE

In order to eliminate the extent of plastic pollution, microorganisms and enzymes from various ecosystems have been isolated and tested for their ability to degrade the different types of PE (Supplementary Table 1). In general, it can be deduced that areas with extensive plastic pollution compel the proliferation of strains capable of degrading polymeric PE resins. Marine environment, which is very burdened concerning plastic pollution, has been the source of bacteria (*Bacillus*, *Pseudonocardia*, *Alcanivorax*, *Marinobacter*, *Arenibacter*, *Brevibacillus* and *Alcanivorax*), as well as some fungi (*Aspergillus tubingensis*, *Aspergillus flavus* and *Zalerion maritimum*) that are capable of polymer degradation and especially PE (Devi et al., 2015; Mohanrasu et al., 2018; Delacuvellerie et al., 2019; Syranidou et al., 2019).

Worms (mealworms/waxworms) have also proven capable of digesting polymer resins, since they are able to adapt their diet to different carbon sources in order to meet their energy needs. There have been some very promising demonstrations of fast degradation of PE by worms. For instance, a waxworm strain had the ability to reduce the weight of HDPE film by 43% after 8 days, while another strain degraded LDPE and pretreated LDPE films by 19% and 56%, respectively, after 7 days (Kundungal et al., 2019a,b). On the other hand, a mealworm managed to mineralize 49% of LDPE foam after a month (Brandon et al., 2018). Gut microbiome seems to change with PE-diet and some of the abundant strains have been isolated and tested against the polymer by themselves, however biodegradation proceeds in a much slower rate and extent (Yang et al., 2014; Brandon et al., 2018; Lee et al., 2020).

Several bacterial and a few fungal strains have been isolated from various sources, such as garbage soil and plastic-waste dumpsite/treatment areas and have been applied for the biodegradation of PE in various forms, however, biodegradation rates are rather slow. For instance, PE film was degraded by *Bacillus aryabhattai* by 23% after 30 days (Devi et al., 2019). LDPE strips are generally degraded faster than pellets with yields reaching 70% after 4 months incubation with an *Enterobacter* sp. (Skariyachan et al., 2016). Comparing HDPE and LDPE strips, the latter are degraded more extensively (58% versus 47%) by a bacterial consortium after almost 5 months (Skariyachan et al., 2018). On the other hand, LDPE powder was degraded by an *Aspergillus terreus* strain by 58.5% after 2 months (Skariyachan et al., 2016; Sangale et al., 2019).

Given the majority of studies on PE degradation include incubation times of over a month, a way to shorten that period could be the pretreatment of materials. Sunlight, temperature, moisture and oxygen supply make resins more vulnerable by breaking down the long polymer chains or forming functional groups, which are better assimilated by microorganisms. While thermal treatment and low irradiation doses have proved to have small impact on the polymer (Novotný et al., 2018), UV irradiation can enhance biodegradation twofold (Hadad et al., 2005). UV-aging in combination with high-temperature treatment (300°C) creates soluble HDPE oligomers that can be assimilated by *Rhodococcus rhodocchrous* almost completely (95%) (Eyheraguibel et al., 2017).

Although different microbes have been studied, their action often follows the same pattern. First of all, considering that PE is a semi-crystalline resin, microorganisms initially degrade the amorphous part of the polymer, this way increasing the crystallinity of the remaining material, making it even more recalcitrant to further degradation (Restrepo-Flórez et al., 2014; Shankar et al., 2019). Moreover, several studies refer that molecular weight has a substantial contribution to the level of degradation. In general, microorganisms firstly assimilate the low molecular weight chains (Mw 4,000–28,000), this way increasing the average molecular weight and then degrade the chains with high molecular weight (Mw > 100,000) at a slower rate (Yoon et al., 2012; Restrepo-Flórez et al., 2014; Shankar et al., 2019). In order to justify the level of biodegradability, several studies analyzed PE with FT-IR analysis, concluding that, due to bacterial action, hydroxyl and carbonyl groups are generated on the surface of polymer (Yang et al., 2014; Azeko et al., 2015; Novotný et al., 2018; Devi et al., 2019; Shankar et al., 2019).

Concerning the mechanism of PE biodegradation by some species, it is shown that the action of extracellular enzymes is crucial. When *Serratia marcescens* subsp. marcescens was grown on LLDPE powder, 36% weight loss was measured after 70 days, while the culture supernatant decreased the powder weight by 31.5% in 28 days (Azeko et al., 2015). Similarly, when *Pseudomonas* sp. E4 was grown on low molecular weight polyethylene (LMWPE) it mineralized up to 28.6% of the carbon after 80 days, secreting alkane hydroxylase (Yoon et al., 2012). Alkane hydroxylase is a key enzyme catalyzing the first step of the alkane degradation, which is the introduction of hydroxyl groups. The respective gene (*alkB*) was cloned in *Escherichia coli* and the recombinant strain was able to turn 19.3% of LMWPE carbon into CO_2_ after 80 days, indicating that *alkB* gene is indeed involved in PE biodegradation (Yoon et al., 2012). *Pseudomonas aeruginosa* E7 was able to mineralize 40.8% of LMWPE, also expressing an alkane hydroxylase gene (Jeon and Kim, 2015).

On the other hand, it is reported that lignin-degrading fungi are able to degrade PE by secreting manganese peroxidase (MnP), whereas surfactant such as Tween 80 slightly increased the degradation level, when added in an MnP system (Iiyoshi et al., 1998). Another enzyme that takes part in lignin biodegradation is laccase, which catalyzes the oxidation of aromatic and non-aromatic compounds (Shankar et al., 2019; Zerva et al., 2019). The role of laccase in degrading long chain alkanes was reported by Sarmah and Rout who measured that the activity of laccase was higher in comparison with peroxidase, when cyanobacteria *Phormidium lucidum* and *Oscillatoria subbrevis* were incubated for 6 weeks using LDPE as sole carbon source (Sarmah and Rout, 2018). When laccase activity was induced in the white-rot fungus *Bjerkandera adusta* TBB-03 by the addition of lignocellulosic material, the strain managed to cause structural changes on HDPE and degrade its amorphous regions (Kang et al., 2019). In a similar manner, by stimulating laccase activity in *Rhodococcus ruber* with the addition of 20 μM Cu^2+^, the weight of PE films was decreased by 2.5%, whereas without the addition of copper the corresponding loss was 1.5%. Meanwhile, when irradiated PE films were treated with laccase the average molecular weight and average molecular number of the polymer reduced by about 20% and 15%, respectively. The minor changes in the molecular dispersity index demonstrate that enzyme acts at the ends of the molecular chains or branches (Santo et al., 2013). To date, the intermediates of PE degradation have not been isolated and identified and the potential of their valorization currently appears limited, however, ultimately, treatments could be developed to afford the release of long-chain alkanes from PE materials, which may prove useful as carbon feedstocks for further valorization.

### Factors Affecting Enzymatic PET Degradation

The biodegradation of PET is the most studied of all the recalcitrant polymers. One of the first reports on microbial PET degradation was in 1982 by surgeons who noticed loss of tensile strength and decrease of molecular mass in bacterial-infected PET implants from patients (Gumargalieva et al., 1982). Since, much progress has been made on the discovery of microorganisms and enzymes that can efficiently hydrolyze PET. Kawai et al. differentiated PET-hydrolyzing enzymes to PET-modifying and PET-degrading enzymes (Kawai et al., 2019). This is an important categorization, as not all PET-hydrolyzing enzymes can be used for the complete degradation of PET into its monomers.

Enzymes that can modify PET’s surface are very useful as catalysts, particularly for the textile industry and enhancing the properties of polyester fabrics (Zhang et al., 2004; Araújo et al., 2007; Kanelli et al., 2015). Surface hydrolysis is the desirable outcome, in order to generate functional groups, without affecting the bulk properties of the polymer.

In the early years of PET-biodegradation research, cutinases were identified as key enzymes that were suitable for this challenging task (Ronkvist et al., 2009), since these enzymes’ natural action is to degrade the plant polyester cutin. Cutinases are serine esterases of the α/β hydrolase family, with their nucleophilic serine exposed to the solvent (unlike lipases) and a flexible active site (Nikolaivits et al., 2018).

Up until 2015 the arsenal of cutinases and other esterases that were able to degrade PET had become richer, comprising of bacterial and fungal enzymes, most deriving from the actinomycete genus *Thermobifida* (Zimmermann and Billig, 2010; Ribitsch et al., 2011; Sulaiman et al., 2012; Kawai et al., 2014; Dimarogona et al., 2015; Supplementary Table 2). In 2016, however, Yoshida et al. reported the discovery of a bacterium originating from a PET-contaminated site, which could assimilate the material after depolymerizing it (Yoshida et al., 2016). The assimilation ability of this bacterium, classified as *Ideonella sakaiensis*, though also contested (Yang et al., 2016), constituted a milestone for PET-biodegradation research. The authors identified the key enzyme that performed the depolymerization of PET, which they named PETase, stating it evolved to act on PET, as a cutinase descendant. The authors report this enzyme to be superior to those already existing, due to its capacity to hydrolyze amorphous PET more efficiently at 30°C (Yoshida et al., 2016).

A lot of effort has been made in order to discover or engineer thermostable PET hydrolases; and for good reason. Due to the nature of its aromatic building block, PET shows inert stiffness, which results in high melting temperature (>230°C) and glass transition temperature (*T*_*g*_) of over 70°C. It has been proven that at temperatures above *T*_*g*_, the amorphous regions of the polymer become very flexible and prone to enzymatic attack (Wei and Zimmermann, 2017). Hence, enzymes which remain stable at high temperatures for long periods of time are considered more suitable for efficient depolymerization. Many ways have been reported in the literature regarding methods to enhance thermal stability of existing PET hydrolases. Dications, such as Ca^2+^ and Mg^2+^ seem to enhance the activity and stability of polyesterases, like the one from *Saccharomonospora viridis* AHK190 (Cut190) and several from *Thermobifida fusca* (TfH, BTA2, Tfu_0882, TfCut1, and TfCut2) increasing the proteins’ melting point (*T*_*m*_) from 1.8–15.2°C (Kawai et al., 2014; Then et al., 2015). Actually, by engineering the ion-binding residues of TfCut2, the need for the addition of ions was lifted and the enzyme presented enhanced thermostability on its own (Then et al., 2015). Further improvement of the thermostability of TfCut2 was achieved by introducing an extra disulfide bridge at the calcium binding site, leading to an impressive increase of *T*_*m*_ by 25°C (Then et al., 2016). Rational structure-based protein engineering has also been applied on IsPETase. A triple mutant was constructed which had a stabilized connecting loop and an extended substrate-binding subsite. These mutations led to a variant with almost 9°C increased *T*_*m*_ (Son et al., 2019).

Apart from thermostability, another crucial factor that can boost enzymatic hydrolysis of PET is product inhibition relief. The most common products of PET hydrolysis are terephthalic acid (TPA) and mono-(2-hydroxyethyl) terephthalate (MHET) (Ribitsch et al., 2011; Sulaiman et al., 2012; Yoshida et al., 2016) or just TPA (Ronkvist et al., 2009; Danso et al., 2018), depending on the hydrolysis mechanism. Accumulating MHET can act as a competitive inhibitor for PET-hydrolases, hindering the overall degradation of the polymer (Barth et al., 2015). Zimmermann group has dealt with this issue using two strategies. One involved the introduction of a carboxyl esterase (TfCa) in the reaction of PET hydrolysis by TfCut2 or LCC, which resulted in twofold increase of released products. TfCa has almost zero activity on PET itself, but can hydrolyze the oligomers released, thus decreasing their inhibitory effect (Barth et al., 2016). The second strategy dealt with mutation of TfCut2’s substrate binding residues in order to resemble LCC. A single variant showed 2.7-fold increased activity on PET, which was attributed to reduced MHET inhibition, based on molecular dynamics studies (Wei et al., 2016).

Research has also focused on the enhancement of PET hydrolases’ activity by increasing the enzymes’ adsorption on the polymer surface. As reviewed by Biundo et al., the activity of PET hydrolases can be enhanced by protein engineering methods either by fusing cutinases with binding modules from other enzymes (e.g., polymer binding module from a polyhydroxyalkanoate depolymerase) or by mutating the surface residues, changing the electrostatic properties of the protein molecules. Alternatively, the addition of hydrophobins (small hydrophobic proteins) in the reaction medium or the fusion of hydrophobins with cutinases can have the same effect increasing the PET-hydrolyzing activity (Biundo et al., 2018). In the same spirit, the group of Miyamoto added surfactants in the reaction medium in order to enhance the adsorption of the enzymes on the polymer’s surface. In the case of IsPETase, an anionic surfactant was used, increasing the overall PET-degradation 120-fold (Furukawa et al., 2018). In the case of TfCut2, a cationic surfactant was used, which increased the degradation of PET films by ca 1.7-fold (Furukawa et al., 2019). The surfactant choice depends on the charge of the enzyme molecule at the given reaction pH and the fact that it does not have adverse effects on the enzyme’s activity or stability.

Using wild-type or engineered cutinases, researchers have managed to degrade PET materials to a considerable degree. As seen in Supplementary Table 2, the most successful results took place at temperatures 63–70°C and pH 7–9 for up to 5 days applying enzyme loading from 0.2 nmol to 1.5 mmol per cm^2^ of material. For all enzymes tested, the highest material weight losses were achieved on low crystallinity PET. Most studies used the same material, which was amorphous PET-GF film (250 μm thick) purchased from Goodfellow (United Kingdom). Different groups have calculated the crystallinity of this material, which varies from 2.3% (Wei et al., 2019) to 9.8% (Barth et al., 2015) and its *T*_*g*_ has been reported to be around 75–76°C (Ronkvist et al., 2009; Kawai et al., 2014). For most enzymatic procedures tested, the weight loss of this material was between 13% and 30%, while exceptionally for some this percentage increased to over 95%. HiC, LCC and TfCut2 (expressed in *Bacillus*) working at 70°C for 4, 2 or 5 days, respectively, resulted in the highest degradation yields (Ronkvist et al., 2009; Shirke et al., 2018; Wei et al., 2019). Another success story for a TfCut2 variant was the degradation of a different amorphous PET film (200 μm thickness and 3–5% crystallinity) which was first coated with an anionic surfactant in order to attract the enzyme molecules. The enzyme achieved full degradation of the film in just 30 h; the fastest reported complete degradation in the literature so far (Furukawa et al., 2019). Apparently, the enzyme concentration increased greatly on the polymer surface, due to the attraction of the surfactant, and the reaction was completed much faster, than in the rest of the cases mentioned above. Recently, a highly thermostable polyethylene terephthalate hydrolase (BhrPETase) was recombinantly expressed in *Bacillus subtilis* and showed a melting temperature as high as 101°C (Xi et al., 2021).

Interestingly, some researchers have tested their enzymes for the degradation of actual post-consumer PET waste (Supplementary Table 2). The problem here is that only some of these materials are characterized in order to make some assumptions regarding the factors affecting their biodegradability. However, Wei and his co-workers performed a very interesting and detailed study for the degradation of two different PET packaging (PET-CP and PET-AP) with low crystallinity (4–6%) in comparison to degradation of the amorphous PET-GF (Wei et al., 2019). An interesting observation was that samples from different part of the packaging showed a vast variation in weight loss (24–57% for PET-CP and 8–50% for PET-AP). This is attributed to the heterogeneity of different parts of the packaging as a result of their thermoforming process leading to local crystallization and discontinuous microstructures. Also, while TfCut2 linearly degraded PET-GF until its ultimate degradation, the degradation of the packaging slowed down (almost stopped) after a certain incubation time. This is attributed to the preferential hydrolysis of the amorphous parts of the material, gradually increasing the crystallinity of the polymer. After all the amorphous parts are degraded, the enzyme very slowly continues the degradation of the crystalline parts. Another important issue raised by this study is the natural aging of the materials at 70°C, which results in increase of crystallinity that hinders the enzyme hydrolysis. Which begs the question: is high temperature appropriate for the enzymatic depolymerization of PET?

Regardless of the enzymatic degradation temperature, a solution to complete depolymerization of crystalline PET could be the combination of chemical with enzymatic treatment. Such process can lead to high purity TPA and EG that could be recovered and used as a feedstock in whole-cell processes for the production of high added-value compounds as described later in the text. There are two relevant examples reported in the literature. One describes the chemical hydrolysis (*T* = 250°C, *P* = 40 bar) of PET fibers that led to 85% TPA and the subsequent enzymatic hydrolysis of remaining oligomers that yielded 97% TPA (Quartinello et al., 2017). A recently proposed alternative is the glycolysis (*T* = 190°C) of crystalline PET granules using a biocompatible chemical catalyst (betain) and the consecutive enzymatic hydrolysis of the resulting BHET using variants of IsPETase and IsMHETase (MHET hydrolase from *Ideonella sakaiensis*) that led to a high concentration of TPA (186.7 mM) (Kim et al., 2021). A combination of ultrasonication and enzymatic catalyzed reaction systems has been recently reported (Kawai et al., 2019). Under optimized conditions, sequential enzymatic-ultrasonic treatment was demonstrated to lead to the release of lower molecular weight products including a higher release of terephthalic acid in solution (Maurya et al., 2020). A significant increase in hydrolysis rate was observed when enzyme-catalyzed hydrolysis was combined with ultrasonication in less crystalline polymers due to more available surfaces for cavitation phenomenon (Pellis et al., 2016). These promising results of combined eco-friendly treatments of crystalline PET materials that lead to high titer and high yield monomer recovery, can be used for the valorization of this plastic.

### Microbial and Enzymatic Degradation of PS

One of the first PS-biodegradation efforts was made in 1979, with no successful results (Kaplan et al., 1979). In this study, 17 species of fungi, five groups of soil invertebrates, and a variety of mixed microbial communities which derived from silt loam, cow manure, activated sludge, or decaying plastics failed to mineralize polystyrene. The scientific group synthesized ^14^C-labeled PS and measured the radioactive CO_2_ released. The failure of this endeavor was mainly based on the incapability of the biochemical catalysts within these microbial communities to biodegrade PS (Kaplan et al., 1979). Although the mechanisms for PS degradation and assimilation may exist in some microorganisms, they have not been studied in depth (Ho et al., 2018; Yang et al., 2018). The inadequate knowledge of these biochemical processes often proves to be a hindrance in increasing biodegradation rates.

In general, PS has been shown to be degraded to a certain extent by bacteria and fungi isolated from various ecosystems. Bacteria belonging to the genera *Paenibacillus*, *Citrobacter*, *Enterobacter*, *Alcaligenes*, *Brevundimonas*, *Bacillus* and *Pseudomonas* have been isolated from the soil and tested for their ability to degrade PS (Atiq et al., 2010; Mohan et al., 2016; Sekhar et al., 2016). The most promising results were for *Bacillus* spp. grown on HIPS films as sole carbon source at 30°C, which reduced polymer mass by 23% after 14 days (Mohan et al., 2016).

In order to enhance PS degradation, Savoldelli et al. (2017) thermally pretreated PS at 240°C and fed the resulting oil to *Pseudomonas putida*. The bacterium broke down polystyrene 98-fold more than the control samples. This impressive degradation enhancement, achieved by the applied pretreatment, can be attributed to the fact that lower molecular weight fragments are created and also cells have more access to the polymeric material (Savoldelli et al., 2017).

There have been numerous reports concerning the ability of different worms to ingest and depolymerize PS. In most cases, this ability has been positively associated with their gut microbiota (Yang et al., 2015b; Peng et al., 2019; Lou et al., 2020). Yang et al., who made the most detailed research concerning the degradation of Styrofoam by larvae of *Tenebrio molitor* concluded that after 16 days 47.7% of ingested PS foam was mineralized, while the rest was egested as fecula and only 0.5% was incorporated into biomass (Yang et al., 2015a).

Fungi have also proven to be good candidates for the biodegradation of PS. *Penicillium variabile* could mineralize a low-MW PS film (15 kDa) 4 times more than a higher-MW one (29 kDa). Furthermore, pretreatment with ozone gas boosted mineralization of PS films from 0.01% to 0.15%. Gel permeation chromatography (GPC) showed a decrease in molecular size, confirming the ability of the fungus to biodegrade PS (Tian et al., 2017). Mushroom *Gloeophyllum trabeum*, which is a lignocellulose degrader, showed that it managed to reduce the average molecular mass (*M*_*n*_) of the soluble polystyrene sulfonate (PSS) up to 80% after 20 days. In an attempt to identify PS metabolites, researches detected 2,5-dimethoxy benzoquinone (2,5-DMBQ) in the culture medium (Krueger et al., 2015). This fungal metabolite can be reduced to the respective hydroquinone creating free radicals that can be transferred to the polymer chain and oxidize it (Fenton-like reaction) (Ali et al., 1993). The authors verified that the decomposition of the polymer was due to the production of 2,5-DMBQ by adding the pure compound to the medium. *G. trabeum* DSM 1398 reduced the average molecular mass (*M*_*n*_) by 50% in 20 days, percentage that increased by 30%, when 2,5-DMBQ was supplementary added. This chemical decomposition of the polymer was confirmed with size exclusion chromatography (SEC), which indicated that chain scissoring happens in random positions across the polymer chain (Krueger et al., 2015).

Mohan et al. (2016) confirmed that extracellular enzymes *Pseudomonas* spp. and *Bacillus* spp. take part in the degradation of HIPS films. The common assumption is that the enzymes that take part in PS biodegradation are oxidases, like peroxidases and laccases (Atiq et al., 2010). However, when *Trametes versicolor* was cultured with PSS as sole carbon source, even though it expressed high laccase activity, it could not degrade the polymer. On the contrary, in the case of commercial laccase from *T. versicolor* in combination with the mediator 1-hydroxybenzotriazole (1-HBT), the system managed to cause 20% reduction in the relative molecular mass (*M*_*n*_) of PSS (Krueger et al., 2015). Furthermore, the pure hydroquinone peroxidase from *Azotobacter beijerinckii* HM121 could degrade PS (*M*_*n*_ 930,000) in a water-dichloromethane biphasic system, releasing products with much lower molecular weight (*M*_*n*_ 350 and 1000), while the aromatic ring was completely degraded (Nakamiya et al., 1997) (Supplementary Table 3).

### Microbial and Enzymatic Degradation of PU

Numerous are the reports on the biodegradation of polyurethanes using bacteria, fungi and even human enzymes (Mahajan and Gupta, 2015), however, given the different structures of the polymer, degradation of one type of PU does not guarantee that the same organism or enzyme will degrade another type of PU, leading to the conclusion that the main factor in PU degradation is the structure of the polymer itself. Probably the most commonly used PU in research is Impranil DLN, a commercial polyester PU (Covestro Ltd.). The exact structure of Impranil DLN is protected (although some researchers have characterized the degradation products) but it is known that it is a polyester type PU with an aliphatic isocyanate making it easily degradable. It is widely used in experiments because it is in a form of colloidal dispersion, making it quite easy to use. The degradation of Impranil DLN is often defined as the “clearing” of the solution, the reduction of absorption OD_600_. Experiments which examined of the degradation products of Impranil DLN have shown that the clearing of Impranil DLN by proteases is not the result of its hydrolytic degradation (Biffinger et al., 2015). Bearing this in mind, any results from studies which had used the clearing of Impranil DLN as the only parameter for PU degradation should be taken into consideration with caution. However, Impranil DLN can be very useful in preliminary screenings as demonstrated by Magnin et al. (2019). The majority of enzymes reported to have PU degrading activity are esterases however only enzymes capable of cleaving the urethane bond can truly be called PUases and useful on a broad range of PU polymers.

Research in the field of PU degradation dates back over 30 years with both whole-cell biocatalysis using bacterial or fungal cells and enzymatic strategies being employed (Mahajan and Gupta, 2015; Howard, 2012). Over the years many bacterial genera have been identified as PU degraders including *Pseudomonas*, *Bacillus*, *Rhodococus*, *Comamonas*, *Acinetobacter*, *Corynebacterium*, and other (Cregut et al., 2013). Probably the best characterized PU degrading enzymes are two “PUases” from *Pseudomonas chlororaphis* (Howard et al., 1999, 2007; Ruiz et al., 1999; Vega et al., 1999) and PU esterase from *Comamonas acidovorans* TB-35. Unfortunately, most of the research on these enzymes was conducted with Impranil which has shown to be fairly easily degradable. A non-exhaustive list of characterized enzymes involved in PU degradation is presented in Supplementary Table 4.

There are also numerous fungal strains reported as PU degraders, but unfortunately not many of their enzymes have been characterized. Fungal strains involved in PU degradation are *Alternaria*, *Aspergillus*, *Cladosporium*, *Cryptococcus*, *Penicillium*, and *Fusarium* species to name but a few (Mahajan and Gupta, 2015). *Aspergillus flavus* strain ITCC 6051 was isolated from soil and showed substantial PU degrading capability. This strain was able to degrade commercial polyester type PU films with up to 60% weight reduction (confirmed using scanning electron microscopy and Fourier transform infrared spectroscopy) after 30 days of incubation (Mathur and Prasad, 2012). More recently degradation of polyester PU was attempted using enriched microbial communities. The results of this study show that microbial consortia grow better than individual members of the community in media with NeoRez R-9637 (a polyester PU) as the sole carbon source, opening up the possibility of novel approaches in PU degradation (Vargas-Suárez et al., 2019).

Commercial enzymes have also been employed in the degradation of PU with the best results obtained by using a combination of an esterase (E3576) and amidase (E4143). This enzymatic cocktail was able to degrade 33% of a PCL polyol-based thermoplastic PU with the esterase cleaving the ester bonds and amidase cleaving the urethane bonds in the polymer (Magnin et al., 2019). Amidases may prove very useful in PU degradation since urethane bond degradation is preferred. A polyamidase from *Nocardia farcinica* fused to a polymer binding module from a PHA depolymerase from *Alcaligenes faecalis* was used in the degradation of commercial PU pellets PU1080 and PU1050 (Bayer), analysis of the degradation products and FTIR confirmed urethane bond cleaving (Gamerith et al., 2016).

Recent studies have made strides in PU degradation product valorization. *Candida antartica* lipase (CALB) was used to degrade PCL/toluene diisocyanate (TDI) based PU with weight loss of 25%. The degradation products obtained were used for the synthesis of second-generation polyester PU polymers (M_*n*_ 24000) (Magnin et al., 2021).

### Biodegradation of PLA

Polylactic acid can be degraded with alkaline treatment, a process that requires large amounts of alkaline reagents to be wasted (Tsuji et al., 2006; Lee and Song, 2011). For this reason, several studies have focused on the discovery of new microorganisms that are able to degrade PLA, despite the fact that their presence in different ecosystems is not as widespread as other microbes that degrade polyesters such as PCL (Pranamuda et al., 1997; Tokiwa et al., 2009). Aerobic bacteria isolated from soil (*Arthrobacter* sp.), water (*Chryseobacterium* sp.), and compost (*Elizabethkingia* sp.) were found to have high hydrolytic activity against PLA films (Walczak et al., 2015). In addition, *Pseudomonas* sp. MYK1 and *Bacillus* sp. MYK2 isolated from a sewage-treatment plant were capable of growing, utilizing PLA films as their sole carbon source (Kim et al., 2017).

It is well-known that biodegradation of PLA resins is almost always due to the production of extracellular enzymes (Jarerat et al., 2006; Butbunchu and Pathom-Aree, 2019). Hence, the cell-free culture medium of *Amycolatopsis orientalis*, which was previously grown in basal medium supplemented with 0.1% (w/v) silk fibroin powder, managed to completely convert PLA powder to L-lactic acid, suggesting that extracellular enzymes may be produced by this bacterium (Jarerat et al., 2006). Another *A. orientalis* that was isolated from soil could reduce PLA film weight by 80% after 8 days (Li et al., 2008). Again, the culture supernatant was incubated with PLA emulsion, which clarified and acidified it, suggesting the action of extracellular enzymes. In order to identify the involved enzymes, the supernatant was fractionated, revealing three enzymes “PLAase” I, II, III, which were classified as serine-like proteases (Li et al., 2008). Two new uncharacterized proteins, which were classified into family V esterases, were isolated from *Alcanivorax borkumensis* and *Rhodopseudomonas palustris* (Hajighasemi et al., 2016). The enzymes managed to degrade emulsified PLA as well as PLA powder and they appear to be specific for poly(DL-lactide). Structural studies of their active site, as well as analysis of hydrolysis products suggest that these enzymes can perform both endo- and exo-esterase cleavage of the polymer (Hajighasemi et al., 2016).

Apart from the aforementioned enzymes, other key players for the depolymerization of PLA are carboxylesterases, cutinases and lipases (Supplementary Table 5; Lee and Song, 2011; Lee et al., 2014; Butbunchu and Pathom-Aree, 2019). From screening environmental metagenomes, two mesophilic carboxylesterases (MGS0156 and GEN0105) belonging to serine α/β hydrolases, were discovered (Hajighasemi et al., 2018). These enzymes were able to hydrolyze synthetic polyesters, including PLA, producing water soluble degradation products such as lactic acid monomers, dimers, and higher oligomers (Hajighasemi et al., 2018). The gene encoding PaE (*PaCLE1*) was cloned in *Saccharomyces cerevisiae* and PLA films were treated with the produced enzymatic solution. SEM analysis revealed structural changes on the PLA film and the amount of total organic carbon (TOC) of the water soluble compounds released, demonstrated that half of the film was degraded (Shinozaki et al., 2013). Furthermore, it is worth mentioning that a study comparing alcalase, esterase and lipase showed that alcalase was the most effective in degrading PLA non-woven fibers. The reduction of PLA weight in the case of alcalase was 25% after 21 days of treatment, yield more than 15 times higher compared to that of the other enzymes (Lee et al., 2014). Concerning Proteinase K, it is one of the first enzymes tested for their ability to degrade polymers, but its application has been gradually reduced, due to its lower activity compared to other PLA-degrading enzymes (Li et al., 2008). Interestingly, when anionic surfactant (sodium dodecyl sulfate-SDS) was added to the enzyme solution, Proteinase K decreased the weight of poly(L-lactide) (PLLA) ultrafine fibers by 80% after 9 h, increasing the degradation rate 10-fold compared to no surfactant addition (Zeng et al., 2004).

Regardless of the type of enzyme used, all of them act in a similar way on the PLA surface, first targeting the amorphous regions of the polymer, which are more vulnerable. This observation is not confined to PLA alone, as the enzymatic decomposition for the majority of polymers, biodegradable or not, follows the same pattern. Furthermore, high crystallinity levels, as well as biaxialy orientated PLA molecules, make the polymer impermeable to enzymatic attack, decreasing the overall biodegradability of PLA samples (Tsuji et al., 2006; Pantani and Sorrentino, 2013).

In order to overcome the limitations of the polymer structure and increase biodegradability, some studies have tested pretreatment methods. Simulated weathering by UV irradiation and water-spray (simulating sunlight and rain, respectively) promoted the cleavage of microfiber, this way increasing the biodegradability rate of non-woven PLA-based mulches up to 72% under composting conditions (Hablot et al., 2014). Moreover, gamma and electron beam irradiation followed by aging of PLA films up to 9 months, resulted in 90% increased mineralization compared to the control samples, under composting conditions (Benyathiar et al., 2016).

### Biodegradation of Polyhydroxyalkanoates

Polyhydroxyalkanoate (PHA) polyesters are bacterially derived so the evolution has also created enzymes able to degrade them (Supplementary Table 6). Chowdhury was the first to isolate PHA-degrading *Pseudomonas* strains and identify the extracellular enzymatic activity responsible for the degradation (Chowdhury, 1963). Since then many PHA-degrading microorganisms (bacteria, yeasts and fungi) have been identified (Roohi, Zaheer and Kuddus, 2018). Due to the ubiquity of such strains, PHAs can be biodegraded in various environments such as river and sea waters, soil and compost (Doi et al., 1996; Volova et al., 2010, 2017; Weng et al., 2011; Boyandin et al., 2013). Dilkes-Hoffman et al. recently performed a very interesting meta-study calculating the degradation rate of PHA products in marine environments, which varied from a few months for bags and straws, to 2-5 years for bottle caps and cutlery (Dilkes-Hoffman et al., 2019).

Microbial genes encoding PHA depolymerases are not exclusive to PHA-producing microorganisms. Knoll et al. created a database containing all the PHA-depolymerase homologs available on databases in 2009 and categorized them in two classes and 7 superfamilies (Knoll et al., 2009). The two classes are intracellular and extracellular depolymerases; this distinction is very important, because these two classes are made to hydrolyze different types of PHAs. Intracellular PHA aka native PHA (nPHA) exists in amorphous granules, while extracellular PHA aka denatured PHA (dPHA), resulting from the cell-lysis of PHA-accumulating bacteria is a partially crystalline (50–60%) polymer (Jendrossek and Handrick, 2002). Furthermore, PHA-depolymerases can be grouped depending on their substrate specificity to short (scl) and medium chain-length (mcl), but there are only a few mclPHA-depolymerases identified so far. PHA-depolymerases are specific for the (*R*)-configuration of the polymers. Studies with oligomeric substrates show that the depolymerase from *Alcaligenes faecalis* could not cleave (*S*)-(*S*) or (*R*)-(*S*) bonds (Bachmann and Seebach, 1999).

The biochemical and structural characteristics of many PHA-depolymerases have been reviewed before (Jendrossek and Handrick, 2002; Roohi, Zaheer and Kuddus, 2018). A very important aspect of this family of enzymes is that they incorporate a substrate binding domain (SBD), which adsorbs on the polymer surface. Hence, hydrolysis by PHB-depolymerases is a two-step reaction, beginning with adsorption, which has its own kinetics and can be a rate-limiting step (Polyák et al., 2019). It should be noted that the SBDs do not show exclusive affinity for the polymers the enzyme can actually hydrolyze (Kasuya et al., 1996). Based on the works of Doi et al., the amount of adsorbed enzyme in more dependent on the amount of ester bonds on the polymer’s surface rather than the hydrophobicity of the surface (Doi et al., 1996; Numata et al., 2009). Additionally, even though crystalline regions of the polymer are degraded much slower by these enzymes, apparently, they are necessary for the enzyme binding, providing a rigid support (Scandola et al., 1997; Focarete et al., 1998).

Crystallinity is very important factor affecting the biodegradability of PHAs. This is why several works have focused on the elucidation of the degradation mechanism of single PHB crystals (Hocking et al., 1996; Iwata et al., 1997; Nobes et al., 1998; Murase et al., 2001; Numata et al., 2009). The results showed that preferential degradation occurs from the crystal edges and ends, while the erosion rate for single crystals depends on the molecular weight of the polymer. The rate was even higher when poly(hydroxybutyrate-*co*-hydroxyvalerate) (PHBV) crystals were tested. In fact, the rate increased for the crystals with higher HV composition (13 mol% versus 6 mol%), possibly due to the higher number of chain folding and ends, allowing more enzyme molecules to bind on the crystal.

Polyhydroxyalkanoate films, on the other side, are composed both of lamellar crystalline and amorphous regions. Melt-crystallized films tend to have higher crystallinity and larger lamellar crystals than solvent-cast films (Numata et al., 2009). As a rule, materials with higher crystallinity are more resistant to enzymatic hydrolysis. Using ^1^H-NMR, it was shown that the amorphous regions of PHB and PHBV films are preferentially hydrolyzed with an exponential rate until a certain point and then the degradation proceeds linearly for both the amorphous and the crystalline regions (Spyros et al., 1997). Furthermore, even though the rate of enzymatic erosion of PHB films with increased crystallinity was decreased, the size of spherulites did not appear to influence the rate of degradation (Kumagai et al., 1992). On the other side, the addition of a small portion of a second monomer in the polymer blend can prove beneficial for the increase of the enzymatic erosion rate. For instance, PHBV films of 41 or 45 mol% HV erode faster than PHB homopolymer of similar crystallinity (Kanesawa et al., 1994). In the case of hydroxyhexanoate (HH) addition, copolymers of P(HB-co-11 mol%HH) eroded over 10 times faster than those of PHB (Abe and Doi, 1999). In the same manner, the erosion rate of PHB copolyesters with lactide (LA) increased over 10 times in the case of P(HB-co-5mol% LA) (Abe et al., 1998).

Other enzymes that can degrade PHAs are lipases, but with a much slower rate (Supplementary Table 6). That can be attributed to the fact that these enzymes have only exo-action on these polymers, while PHA-depolymerases also act in an endo-manner (Shang et al., 2012; Li et al., 2019; Mandic et al., 2019). However some microbial lipases can hydrolyze mclPHAs (Mukai et al., 1993), while there are only a few characterized mclPHA-depolymerases (Schirmer et al., 1993; Rhee et al., 2006; Gangoiti et al., 2010; Gangoiti et al., 2012; Santos et al., 2013; Martínez et al., 2015).

### Biodegradation of Poly(Butylene Succinate) and Copolymers

Poly (Butylene Succinate) (PBS) and its copolymers are degraded by various microorganisms in different environments. It has been found that bacteria (Lee and Kim, 2010), fungi (Suzuki et al., 2014; Hu et al., 2016) and yeasts (Thirunavukarasu et al., 2016) possess all the necessary mechanisms for PBS degradation, which are mainly based on the secretion of extracellular enzymes (Xu and Guo, 2010). However, the biodegradation of PBS is a complex process affected by multiple factors. As with all polymers, the lower degree of crystallinity, as well as increased hydrophilicity and surface area of the polymer specimen are significant factors which increase the rate of biodegradation (Li et al., 2007; Lee et al., 2008; Xu and Guo, 2010; Gigli et al., 2016).

The mechanism of PBS hydrolysis begins with the non-specific adsorption of the enzyme on polymer surface (Mochizuki et al., 1997; Taniguchi et al., 2002; Lindström et al., 2004). Most of the studies mention that enzymatic degradation catalyzed by lipase follows an exo-type mechanism, according to which the enzymes hydrolyze the ester bond, causing surface erosion (Taniguchi et al., 2002; Li et al., 2007). Meanwhile, enzymatic hydrolysis is induced by a non-specific, non-enzymatic, endo-scission, caused by water molecules and catalyzed by the free carboxyl groups of PBS, releasing low molecular weight oligomers, which are subsequently hydrolyzed to dimers and monomers by the enzyme (Taniguchi et al., 2002; Honda et al., 2003). The end products of biodegradation have been identified as 1,4-butanediol, succinic acid, 4-hydroxy-butyl succinate, di(hydroxy-butyl) succinate and hydroxybutyl disuccinate (Taniguchi et al., 2002; Lindström et al., 2004; Lee et al., 2008; Sato et al., 2017).

The enzymatic hydrolysis of PBS can be catalyzed by lipases, esterases, cholesterol esterases and cutinases (Supplementary Table 7). Especially, lipases derived from *Pseudomonas* sp. have been well studied for their effectiveness in degrading PBS (Gan et al., 2001; Taniguchi et al., 2002; Honda et al., 2003; Tserki et al., 2006). Poly(butylene succinate-*co*-L-lactate) (PBSL - 97/3 mol%) and pure PBS films were treated with a lipase derived from *Pseudomonas cepacia*, resulting in a weight loss greater than 80% after 14 days (Taniguchi et al., 2002). Moreover, when a lipase from the same bacterium was added to organic solvent (chloroform), it reduced the average molecular weight of poly(butylene succinate-co-butylene adipate) (PBSA, 90/10 mol%) and poly(butylene succinate-co-hexane succinate) (90/10% mol) films by 70 and 80% after 60 h, respectively (Li et al., 2015). Another lipase derived from a *Pseudomonas* sp. decreased the weight of PBSA (90/10 mol%) films by 96% within 10 days (Tsutsumi et al., 2003). In general, it can be concluded that PBS-based polymers, with long chain co-monomers or with a co-monomer ratio of less than 10%, are degraded faster than PBS homopolymer, mainly due to the lower degree of crystallinity (Gan et al., 2001; Li et al., 2015; Gigli et al., 2016).

Although lipases degrade a wide variety of PBS based-polymers, cutinases derived from the fungus *Fusarium* sp. can achieve higher degradation rates than lipases (Supplementary Table 7). For example, a cutinase from *Fusarium solani* completely degraded pure PBS films after 26 h (Shi et al., 2019). Additionally, another cutinase from a *Fusarium* sp. completely degraded PBS films with average molecular weight (Mn) 1.5-3.7 × 10^4^ in less than 10 h (Hu et al., 2016; Pan et al., 2018). In contrast to lipases, cutinases probably cause endo-type scission of PBS polymer, producing succinic acid monomer and oligomers such as di(hydroxy-butyl) succinate and hydroxybutyl disuccinate, which are subsequently hydrolyzed into monomers and dimers (Shi et al., 2019).

### Microbial and Enzymatic Degradation of PCL

PCL degradation has been reported to date by a variety microorganisms isolated from soil, including both aerobic and anaerobic bacteria (*Bravundimonas* sp., *Clostridium* sp., *Streptomyces* sp.) (Benedict et al., 1983; Abou-Zeid et al., 2001; Nawaz et al., 2015; Mandic et al., 2019), fungi (*Penicillium oxalicum*) (Li et al., 2012) and yeasts (*Cryptococcus laurentii*) (Benedict et al., 1983). Bacteria isolated from sea water, able to grow under mild (*Streptomyces* sp.) or extreme temperature and pressure conditions (*Pseudomonas* sp.), could degrade and consequently assimilate PCL in laboratory tests (Sekiguchi et al., 2011; Almeida et al., 2019). All these isolates confirm that microorganisms able to degrade PCL are distributed in different ecosystems (Tokiwa et al., 2009).

Despite the fact that PCL-degrading microorganisms are ubiquitous, PCL degradation is strongly influenced by environmental conditions. For instance, under anaerobic conditions and in presence of a redox indicator, a bacterium belonging to the genus *Clostridium* decreased the weight of PCL strips by 7.6% after 10 weeks (Abou-Zeid et al., 2001). On the other hand, under modeled composting conditions indigenous microorganisms augmented by *Streptomyces* sp. BV315 decreased the weight of PCL films up to 18% after 4 weeks (Mandic et al., 2019). Apart from growth conditions, pretreatment methods enhance biodegradation of the polymer, making the substrate more vulnerable. For example, bacterium *Alcaligenes faecalis* completely degraded UV-treated PCL films within 68 days, whereas without UV treatment almost no weight loss was observed (Khatiwala et al., 2008).

The microbial degradation of PCL requires the secretion of extracellular enzymes. The production of extracellular enzymes was assumed when the fungus *Penicillium oxalicum* DSYD05 completely assimilated PCL films after 9 days, when grown in mineral medium. Meanwhile, the supernatant was collected and incubated with PCL suspension, thereby reducing its turbidity (Li et al., 2012). Following the same experimental procedure, *Brevundimonas sp.* strain MRL-AN1 reduced the weight of PCL film by 80% in 10 days, while the supernatant could clarify PCL emulsion (Nawaz et al., 2015). Concerning the mechanism of enzymatic degradation, enzymatic hydrolysis takes place on the polymer surface, because hydrophilic enzymes cannot diffuse into a hydrophobic plastic, such as PCL (Azevedo and Reis, 2005). The PCL degradation ends with the creation of products, such as 6-hydroxycaproic acid (Li et al., 2012), while new functional groups, such as –OH, –COO^–^, –COOH are being generated (Sivalingam et al., 2003). Again, crystallinity is a very important factor affecting the enzymatic hydrolysis of PCL as well (Tokiwa et al., 2009; Ping et al., 2017). Specifically, as with all semi-crystalline polymers, in low crystallinity level (approximately 24%), enzymes degrade the amorphous part, while in higher crystallinity (> 40%) degradation takes place in both amorphous and crystal region (Gan et al., 1997; Khatiwala et al., 2008; Sekosan and Vasanthan, 2010; Li et al., 2012). Additionally, the degradation rate is increased when the crystallinity level is low. For example, the weight of PCL pellets was decreased by a commercial *Pseudomonas* lipase by 80% and 20%, when the initial crystallinity was 24% and 45%, respectively (Sekosan and Vasanthan, 2010).

The most well-studied enzymes for the hydrolysis of PCL are lipases, esterases and cutinases (Sivalingam et al., 2003; Pastorino et al., 2004) (Supplementary Table 8). Three commercial lipases derived from *Candida rugosa*, *Mucor miehei* and *Rhizopus delemar* were tested for their effectiveness in PCL degradation. PCL was hydrolyzed in different toluene/water ratios by lipases, concluding that an essential amount of water promotes enzymatic activity, while degradation occurs in organic solvent. From the three enzymes tested, only lipase from *Mucor miehei* reduced the average molecular weight of PCL up to 74% at 40°C within 24 h (Pastorino et al., 2004). In addition, three purified lipases derived from different sources, were incubated with PCL films at 37°C for 100 h. *Pseudomonas* lipase was the only enzyme that completely degraded PCL films, unlike the other enzymes, which slightly reduced film weight (Gan et al., 1997). Similarly, by measuring the turbidity reduction, a purified lipase derived from *Bacillus subtilis* degraded PCL emulsion within 10 min, whereas a lipase and an esterase from *Burkholderia* sp. and *Ophiostoma piceae*, respectively, slightly reduced the PCL initial concentration (Lin et al., 2019).

Apart from esterases and lipases, a cutinase-like enzyme named PaE from the yeast *Pseudozyma antarctica* JCM 10317 was used to treat PCL films, resulting in 61.5% biodegradation (Shinozaki et al., 2013). Another study also examined the activity of cutinases derived from the fungi *Fusarium solani* and *Aspergillus fumigatus*. The weight of PCL films treated with the cutinase of *F. solani* reduced by 44.3% at 40°C after 6 h, while the *A. fumigatus* cutinase completely degraded the PCL films, under the same conditions (Ping et al., 2017).

## Valorization of Monomers and Other Plastics Degradation Products

The concept that goes beyond biological degradation of plastic waste and covers monomer recovery and utilization for new materials is coined as bio-upcycling. This concept has been overviewed (Blank et al., 2020; Ru et al., 2020) and recently demonstrated by Tiso et al. (Tiso et al., 2020) in the case of PET.

### Monomers From Plastic Degradation and Their Recovery

Despite the recognized need to capture and viably utilize the products of biological degradations of plastics, very little has been done on the downstream processing including recovery and separation of monomers.

Degradation pathways and corresponding monomeric products have been proposed for all major types of plastics and bioplastics (RameshKumar et al., 2020; Ru et al., 2020). Specific metabolic pathways also exist in number of microorganisms to convert these into valuable bio-chemicals and materials, such as succinic acid, rhamnolipids and PHAs (Ru et al., 2020).

Monomers of PET such as BHET, TPA and EG can be obtained via enzymatic treatments (Yoshida et al., 2016; Carniel et al., 2017), glycolysis (Imran et al., 2010), or alkaline hydrolysis (Karayannidis et al., 2002). Recently a wider selection of PET-related building blocks has been synthetized and characterized (Djapovic et al., 2021). The only industrially relevant bio-process was achieved by Tournier et al. employing enzymatic degradation of waste PET bottles and obtaining 16.7 g/L/h of terephthalate that was subsequently used for the synthesis of virgin polymers (Tournier et al., 2020). Also regarding PET, a strain of *Pseudomonas putida* was grown directly on PET enzymatic hydrolysate to obtain PHA (Tiso et al., 2020).

Microbial PS metabolites that have been identified so far are 2-phenyl ethanol, 1-pheny-1,2-ethanediol, phenylacetaldehyde and styrene oxide (Atiq et al., 2010; Mohan et al., 2016; Sekhar et al., 2016), which are styrene metabolites and can be assimilated by microorganisms. Especially for the styrene assimilation, a number of intracellular enzymes are necessary. The oxidation of the vinyl side chain of styrene is catalyzed by styrene monooxygenase enzyme resulting in the formation of styrene epoxide. Afterward, styrene oxide is isomerized either to phenylacetaldehyde by styrene oxide isomerase or to 2-phenylethanol. Phenylacetaldehyde is subsequently converted to phenylacetic acid through the action of either an NAD + or phenylacetaldehyde dehydrogenase. Then, phenylacetic acid is converted to phenylacetyl-CoA, which undergoes numerous aerobic enzymatic reactions, before entering the tricarboxylic acid (TCA) cycle (Mooney et al., 2006).

Lactic acid (LA; 2-hydroxy propionic acid) is perhaps the most widely occurring carboxylic acid in nature and is the one monomer which can be produced from a varied range of carbohydrate-rich renewable resources using fermentation technology (Wee et al., 2006; Bishai et al., 2013; Ghaffar et al., 2014; Zaaba and Jaafar, 2020). After fermentation, lactic acid must be recovered from the broth and purified to meet its final specifications. Depending on the raw materials, microorganisms, and media used, the typical recovering steps are: (1) removal of microbial biomass, (2) sulfuric acid treatment, (3) removal of gypsum to recover crude lactic acid, and (4) purification of lactic acid by ion exchange chromatographic resins to produce food-grade lactic acid, and/or by distillation to produce polymer (or heat-stable) grade lactic acid free of any protein, sugar and most other impurities, as the purity of lactic acid or lactide strongly affects the characteristics of PLA (Jem and Tan, 2020). Integrated approaches to efficiently generate and regenerate PLA are still needed.

So far, the only viable degradation and subsequent valorization of plastics was obtained through pyrolysis of polymers. By pyrolysis of PET, a solid fraction consisting of 72% TPA monomers and 26% TPA oligomers was obtained (Kenny et al., 2008). Styrene oil consisting of 82.8% styrene was obtained by pyrolysis of PS (Ward et al., 2006). PE pyrolysis wax consisting of aliphatic hydrocarbons ranging from C8 to C32 was obtained by pyrolysis of postconsumer PE (Guzik et al., 2014).

Overall, in the case of polyesters, mechano-green-chemical treatments have the potential to lead to monomer streams of sufficient purity to enable repolymerization as virgin polyester as in the case of virgin PET to virgin PET circularity. In the case of mixed polyesters or PE and PP, further biocatalytic treatment has the potential to induce further depolymerization leading to output monomer, mixed monomer and oligomer feedstocks which can be used for the fermentation of biopolymers such as PHAs.

### (Bio)Upcycling Processes

Despite the fact that numerous bio-upcycling routes have been proposed for the generation and regeneration of new valuable chemicals and materials, the examples of these processes are still scarce.

The pyrolysis products from the PS and PET were directly used as carbon source for the production of mclPHAs by variety of wild type *Pseudomonas* spp. and moderate to good yields were obtained using optimization of fermentation strategies (Goff et al., 2007; Kenny et al., 2008; Nikodinovic-Runic et al., 2011). PE pyrolysis wax consisting of aliphatic hydrocarbons ranging from C8 to C32 was used by *Pesudomonas areuginosa* GL-1 and *P. oleovorans* B-14682, in the presence of a biosurfactant, for the production of mclPHA (Guzik et al., 2014). More recently non-oxygenated PE wax was used as a sole carbon and energy source for the production of PHB by *Cupriavidus necator* H16 (Johnston et al., 2017).

Apart from the obvious use in the production of new PET, degradation products can be used as reagents to obtain novel compounds. BHET obtained by glycolysis and purified can be converted to quaternary ammonium compounds, that can be used as softeners for cotton fabrics (Shukla et al., 2008). TPA can be converted into a range of aromatic and aromatic-derived added value compounds such as gallic acid (used in the pharmaceutical industry to produce an antibacterial agent, trimethoprim, and as an antioxidant- propyl gallate), pyrogallol (used as an antioxidant in the oil industry), catechol, muconic acid (used in the chemical industry to produce adipic acid, which is widely used to produce plastics), and vanillic acid (used as the direct precursor of vanillin in the pharmaceutical industry). TPA was converted using *E. coli* strains metabolically engineered to contain the TPA degradation pathway (Kim et al., 2019). In the same fashion EG was used to produce glycolic acid (used as an exfoliant in the cosmetic industry). The use of TPA in the production of 1,4-dialkylbenzenes, used as intermediates in organic synthesis, was proven to be superior to classic methods of production (Bramborg and Linker, 2010).

The PS monomer, styrene, can be biologically converted to 3-vinylcatechol (used in the synthesis of polymers, dyes, and pharmaceutical products) by *R. rhodochrous* NCIMB 13259. Using styrene monooxygenase, styrene can be converted to styrene oxide (used as a reactive plasticizer or diluent for epoxy resins) in an enantio-selective manner (Mooney et al., 2006).

Polyurethanes degradation products can also be utilized by microorganisms and converted into value added products. A defined microbial consortium was used to convert a blend of hypothetical PU degradation products (adipic acid (AA), 1,4- butanediol (BDO), ethylene glycol (EG) and 2,4 TDA) to rhamnolipids. The consortia consisted of *Pseudomonas* sp. TDA1 and three engineered *P. putida* KT2440 strains (utilizing EG (Li et al., 2019), BDO (Li et al., 2020) and AA). The *Pseudomonas* strains were transformed with plasmid pPS05 (containing *rhlA* and *rhlB* genes) for rhamnolipid synthesis. Due to the fact that TDA inhibited the growth of other consortium members, TDA had to be extracted for the efficient rhamnolipid production. Even after TDI extraction, rhamnolipid production of the consortium was lower than that of each individual strain on the preferred monomer (Utomo et al., 2020).

Lactic acid (LA) has numerous uses in the food, pharmaceutical, leather tanning industries, and can be converted to various chemicals with different applications (Komesu et al., 2017). Since it contains hydroxyl and carboxylic functional groups it can be converted to a large number of commodity chemicals trough different reactions. Acrylic acid (use in polymeric flocculants, dispersants, coatings, paints, adhesives, and binders for leather, paper and textile) and pyruvic acid (used as a starting material for the synthesis of many drugs and agrochemicals and, presently, in the food industry as a fat burner) are obtained by dehydration of LA. By condensation 2,3- pentanedione (used as a flavoring ingredient) can be produced. Propanoic acid (used in the production of cellulose plastics, herbicides, and perfumes, and propionic salts are used as food preservatives) and 1,2- propanediol (used as a de-icing fluids, antifreeze, for the production of unsaturated polyester resins, and in the production of drugs, cosmetics, and foods) obtained by reduction, acetaldehyde by decarboxylation and ethyl lactate (used as a solvent and oligomers of ethyl lactate can be used as plasticizers) by esterification with ethanol (Fan et al., 2009). These chemicals are traditionally produced from fossil fuel feedstock so conversion of lactic acid provides an environmentally friendly alternative in the production of commodity chemicals. A detailed review on the conversion of LA to commodity chemicals has been made by Maki-Arvela et al. (2014).

## Concluding Remarks and Future Prospects

‘Biocyclable’ plastics, where post-consumer plastics are bio-depolymerized and subsequently bio-repolymerized, presents the archetypal target for next generation sustainable plastics. Biomimetic strategies and bio-based approaches, which mirror nature and endeavor to address the innate bio-inert properties of plastic, are being newly developed and applied to waste plastic. The overall aim of these approaches is to biotechnologically depolymerize waste plastics funneling the output monomers and oligomers for value added product production in the drive to generate alternative bioplastic products in a sustainable circular process (Figure 2).

All of the different components necessary to deliver the imperative task of completing the life-cycle of plastics are available and ready to be enhanced, honed and activated to transform how we live with plastic. Most critical is the need to merge and integrate processes and technologies from across the different disciplines to progress convergent mechanisms as demonstrated in nature. It is clear that, while important achievements have been made within specific disciplines, this is generally using singular technologies or the combination of a small number of related technologies, rather than encompassing cross disciplinary methodologies. In the case of mechano-thermo-photo irradiative and green chemical approaches, one or a combination of two dedicated techniques have been successfully applied to achieve important breakthroughs such as chain scissions and significant molecular weight reductions. However, these advancements have only rarely been explored in conjunction with enzymatic or microbial activities. A combination of ultrasonication and enzymatic catalyzed reaction systems has been recently reported, however, further evaluation and tailoring of mechano-thermo-photo-irradiative induced amenability of plastics to biocatalytic processes is essential in the progression toward plastics circularity. Life cycle analysis data will be instrumental in establishing the most sustainable mechano-green-chemical-biocatalytic treatment routes to achieve optimal sustainable circularity and will be essential in guiding the development of these approaches to achieve maximal regenerative process yields which are industrially scalable and present the lowest carbon footprint.

Wild-type microorganisms and communities from the natural habitats are immensely diverse and their formidable potential for development and harnessing for the controlled biodegradation of waste plastic materials is clearly evident. Microplastics that have been in the environment over long durations already exhibit enrichment of specific bacterial communities. The exploration of enzymatic activities and enzymatic synergies for biotechnological processes is rapidly gaining recognition. On the other side, extremophilic microorganisms exist, with a number of proven strategies that allow them to survive in extreme conditions including high salt concentrations and both high and low temperatures. Systematic application of selected combination and microbial and enzymatic approaches presents a gateway to regenerative plastic circularity. The progression to deal with more complex plastics such as mixed multilayers is feasible through dedicated exploitation of microbiological diversity and novel enzymatic activities to catalyze the bioconversion of non-polyester plastics such as PE and PP. These novel enzymatic activities can be discovered through multi-omic techniques leading to redox biocatalysts, such as peroxidases, multicopper oxidases and monooxygenases, capable of incorporating oxygen atoms to the inert carbon polymer backbone, allowing the desired biological degradation.

Achievement of controlled biodegradation is a vital prerequisite in avoiding resource loss, which is an important limitation of methods such as biodegradable polymer composting. Channeling mechano-thermo-photo-irradiative-biocatalytic depolymerized output monomers and oligomer molecules as feed stocks ready for repurposing within continuous loops is integral in completing the circularity template. These loops and cycles are defined by their capacity to yield products and outputs with value at least equivalent to the input plastics, rather than through downcycling or cradle to grave avenues: the case in the majority of solely mechano-thermo approaches which generate products such as plastic composite outdoor furniture and energy extraction. Continuous loops such as conversion to biodegradable polymers or entry into closed loops such as PET virgin to virgin repolymerization, provide continuous regenerative circularity akin to cycles manifested in nature. In the case of conversion to biopolymers such as PHA’s, further scrutiny of the structure-performance relationship is required accompanied with the advancement of bio-chemosynthetic and polymer processing approaches in order to obtain their full potential as biodegradable polymers with equivalent performance to petroleum-based counterparts such as PE and PP. As we advance with the support of nature’s guidelines, adopting regenerative principles, achievable through in-tandem cross-disciplinary progress, the realm of resource neutral, low carbon and energy plastics circularity beckons, impelling and compelling us to bring it to reality.

## Author Contributions

JN-R and MBF generated the idea and designed the project. ET and RB contributed to the analysis and discussion. GT, BP, and MA prepared the figures and tables. EN and MBF drafted the manuscript and all co-authors reviewed and edited the final version of the manuscript. All authors contributed to the article and approved the submitted version.

## Conflict of Interest

The authors declare that the research was conducted in the absence of any commercial or financial relationships that could be construed as a potential conflict of interest.
