# Supplementary material for: Progressing Plastics Circularity: A Review of Mechano-Biocatalytic Approaches for Waste Plastic (Re)valorization
Source: Front Bioeng Biotechnol. 2021 Jun 22;9:696040. doi: 10.3389/fbioe.2021.696040 (PMC8260098; doi:10.3389/fbioe.2021.696040)
Supplement: Supplementary file 1 [file Data_Sheet_1.PDF]

*Supplementary Material*

# Progressing Plastics Circularity: A Review of Mechano-Biocatalytic Approaches for Waste Plastic (Re)valorization

**Efstratios Nikolaivits[1], Brana Pantelic[2], Muhammad Azeem[3], George Taxeidis[1], Ramesh Babu[4], Evangelos Topakas[1], Margaret Brennan Fournet[3]*, Jasmina Nikodinovic-Runic[2]***

[1] Industrial Biotechnology & Biocatalysis Group, Biotechnology Laboratory, School of Chemical Engineering, National Technical University of Athens, Athens, Greece

[2] Eco-Biotechnology & Drug Development Group, Laboratory for Microbial Molecular Genetics and Ecology, Institute of Molecular Genetics and Genetic Engineering, University of Belgrade, 11000 Belgrade, Serbia

[3] Athlone Institute of Technology, Dublin Road, Athlone, Co. Westmeath, Ireland

[4] AMBER Centre, CRANN Institute, School of Chemistry, Trinity College Dublin, Dublin, Ireland

*** Correspondence:**
Margeret Brennan Fournet
mfournet@ait.ie

Jasmina Nikodinovic-Runic
jasmina.nikodinovic@gmail.com, jasmina.nikodinovic@imgge.bg.ac.rs

## Supplementary Tables

**Table S1.** Enzymes involved in polyethylene (PE) degradation. Reaction conditions and effect on specific materials are reported for each enzyme.

| Enzyme | Origin | Material | Experimental Conditions | Results | Ref |
|---|---|---|---|---|---|
| Alkane hydroxylase | *Pseudomonas aeruginosa* E7 | LMWPE powder<br>Mw 1.700 | Compost, 37°C, 80 days<br>3.5 g LMWPE blended with 200 g (wet weight) sterilized compost.<br>Bioaugmentation with *P. aeruginosa* E7 | 40.8%<br>mineralization | (Jeon & Kim, 2015) |
| Alkane hydroxylase | *Pseudomonas* sp. E4 | LMWPE[a]<br>Mw 1.700 | Compost, 37°C, 80 days,<br>PE 5 wt% of compost.<br>The mixture was inoculated with *Pseudomonas* sp. E4 | 28.6% mineralization | (Yoon et al., 2012) |
| Laccase | *Rhodococcus ruber* | LDPE film<br>0.92 g/ $cm^3$<br>Mw 191,000<br>3 cm x 3 cm<br>0.2 mm thick | 50 ml synthetic medium, 20µM $Cu^+$, 30°C, 30 days<br>300 mg PE films<br>Bioaugmentation with *Rhodococcus ruber* | 2.5% weight loss | (Santo et al., 2013) |

[a]Low-Molecular-Weight Polyethylene

**Table S2.** Enzymes involved in polyethylene terephthalate (PET) degradation. Reaction conditions and effect on specific materials are reported for each enzyme.

| Enzyme | Origin | GenBank/<br>PDB ID | Material | Reaction conditions | Weight loss (%) | Ref. |
|---|---|---|---|---|---|---|
| TfH | *Thermobifida. fusca* | ALF04778.1 | PET-G<br>Beverage bottle films<br>$T_g$=75 °C<br>12 mm diameter | 55 °C, 0.5 mL 0.1 M Phosphate pH 7, 3 weeks with buffer change/enzyme addition<br>(4-5 mg film/mL reaction)<br>0.8 nmol enzyme/mg film/week or 16 nmol enzyme/cm$^2$ film/week | 54.2 | (Müller et al., 2005) |
| | | | PET-B<br>(9-10% crystallinity-BASF, DE)<br>$T_g$ =80 °C<br>12 mm diameter | | 13.1 | |
| TfH | | ALF04778.1 | PET-GF* film (Goodfellow, UK)<br>250 µm thick<br>(crystallinity 2.3% by (Wei *et al.*, 2019))<br>(crystallinity 9.8% by (Barth *et al.*, 2015)) | 65 °C, 1.5 mL 1 M Tris pH 8.5, 10 mM $CaCl_2$, 2 d<br>(108 mg film/mL reaction)<br>2.5 nmol enzyme/mg film or 0.4 nmol enzyme/cm$^2$ film | 13 | (Then et al., 2015) |
| TfCut2 | | 4CG1 | | | 12.6 | |
| TfCut2 variant (D204C-E253C-D174R) | | | | 70 °C, 1.5 mL 0.5 M HEPES pH 8, 2 d<br>(30 mg film/mL reaction)<br>35 µmol enzyme/mg film or 0.5 mmol enzyme/cm$^2$ film | 25 | (Then et al., 2016) |
| TfCut2 expressed in *Bacillus megaterium* | | | | 70 °C, 1.8 mL Phosphate pH 8, 5 d<br>(25 mg film/mL reaction) | 97 | |

| | | | | | | |
|---|---|---|---|---|---|---|
| | | | | 0.03 nmol enzyme/mg film or 1 nmol enzyme/cm$^2$ film | | |
| | | | PET-CP, post-consumer Carton Pack (Carton Pack Srl, Rutigliano, Italy)<br>Crystallinity 4-6% | 70 °C, 1.8 mL Phosphate pH 8, 7 d<br>(17 mg film/mL reaction)<br>0.05 nmol enzyme/mg film or 1 nmol enzyme/cm$^2$ film | 23.9-56.6 | (Wei et al., 2019) |
| | | | PET-AP, post-consumer Agripack (Groupe Guillin, Ornas, France)<br>Crystallinity 4-6% | 70 °C, 1.8 mL 1 M Phosphate pH 8, 7 d<br>(15 mg film/mL reaction)<br>0.04 nmol enzyme/mg film or 1 nmol enzyme/cm$^2$ film | 8.2-50.5 | |
| TfCut2 | | | Low crystallinity PET sheets (NOACRYSTAL-V)<br>200 μm thick<br>3-5% crystallinity<br>Coated with $C_{12}$-$N(CH_3)_3^+$ surfactant | 65 °C, 0.5 mL 0.15 M Bicine pH 9, 10 mM $CaCl_2$, 2 d<br>(15 mg film/mL reaction)<br>0.07 nmol enzyme/mg film or 0.8 nmol enzyme/cm$^2$ film | 30 | (Furukawa et al., 2019) |
| TfCut2 variant (G62A/F209A) | | | | 65 °C, 1 mL 0.15 M Bicine pH 9, 10 mM $CaCl_2$, 1.25 d<br>(7.5 mg film/mL reaction)<br>0.13 nmol enzyme/mg film or 1.7 nmol enzyme/cm$^2$ film | 100 | |
| LCC | Uncultured bacterium | 4EB0 | PET film from plastic package | 50 °C, 1 mL 0.5 M Tris pH 8, 1 d<br>(20-25 mg film/mL reaction)<br>8 pmol enzyme/mg film or 3.6 nmol enzyme/cm$^2$ film | 6.5 | (Sulaiman et al., 2012) |

| | | | | | | |
|---|---|---|---|---|---|---|
| | | | PET-GF* film (Goodfellow, UK)<br>250 µm thick<br>crystallinity 7% | 70 °C, 3 mL 0.5 M HEPES pH 8, 2 d<br>(30 mg film/mL reaction)<br>3 mmol enzyme/cm$^2$ film | 95 | (Shirke et al., 2018) |
| LCC-glycosylated | | | | | 95 | |
| Cut190* | *Saccharomonospora viridis* | 4WFK | PET-S from package<br>600 µm thick | 63 °C, 1 mL 0.1 M Tris pH 8.2, 50 mM $CaCl_2$, 24% glycerol, 3 d<br>(20-25 mg film/mL reaction)<br>11 nmol enzyme/mg film | 27 | (Kawai et al., 2014) |
| | | | PET-GF* film (Goodfellow, UK)<br>250 µm thick<br>$T_g$=76 °C | | 14 | |
| HiC | *Humicola insolens* | 4OYY | PET-GF* film (Goodfellow, UK)<br>250 µm thick<br>(crystallinity 7%)<br>$T_g$=75 °C | 70 °C, 3 mL 1 M Tris pH 7.5, 10% glycerol, 4 d<br>13 nmol enzyme/ cm$^2$ film | 97 | (Ronkvist et al., 2009) |

[a] Enzyme concentration per film area is calculated considering that both sides of the film are accessible to the enzyme

**Table S3**. Enzymes involved in polystyrene (PS) degradation. Reaction conditions and effect on specific materials are reported for each enzyme.

| Enzyme | Origin | Material | Experimental Conditions | Results | Ref |
|---|---|---|---|---|---|
| Hydroquinone Peroxidase | *Azotobacter beijerinckii* HM121 | Dissolved PS (Aldrich Chem. Co. Inc.) Mn 930,000 | 0.4 ml of water, 10 mM hydrogen peroxide, 10 mM tetramethylhydroquinone, 100 mM potassium phosphate buffer pH 7.0, 30°C, 10 min 0.4 ml of dichloromethane containing 2 g/L of polystyrene. 2.4 U/mL of hydroquinone peroxidase (1.0 mg/mL protein) | Mn reduced to 350 and 1,000 | (Nakamiya et al., 1997) |
| Lacasse | *Trametes versicolor* Commercial laccase (Sigma-Aldrich) | PSS (Sigma-Aldrich) Mw 70,000 Da | 28°C, 5 ml of 50 mM malonate buffer pH 4.5, 0.25 mM of redox mediator 1-HBT, 4 days 1 mg PSS / mL reaction 20 mg protein-10 U (0.5 U / mg protein) | Mn reduced to 56,000 Da | (Krueger et al., 2015) |

**Table S4.** Enzymes involved in polyurethane (PU) degradation. Reaction conditions and effect on specific materials are reported for each enzyme.

| Enzyme | Origin | GeneBank/ PDB ID | Material | Experimental Conditions | Results | Ref |
|---|---|---|---|---|---|---|
| PU esterase | *Comamonas acidovorans* TB-35 (*Delftia acidovorans* | AB009606.1/ Q9WX47 | Polyester PU (poly(diethylene glycol adipate) and 2,4 TDI) | PU blocks (4x4x1mm) incubated with cell free extract of transformed *E. coli* for 48 h at 30°C | 50 µmol/ mg protein/ 48 h diethylene glycol, adipic acid, trimethylol propane and 2,4-diaminotoluene released | (Nomura et al., 1998) |
| Polyurathenase lipase A (PulA) | *Pseudomonas fuorescens* | AF144089.1/ Q9LAB9 | Impranil DLN | Impranil in agar plates incubated at 30°C | Clearing halos were observed | (Vega et al., 1999) |
| Polyurethanase esterase A (PueA) | *Pseudomonas chlorapis* | AF069748.1/ Q9X3C0 | Impranil DLN | Radial diffusion assay with purified enzyme on agar plates with 1.5% v/v Impranil DLN incubated for 6 h at 26°C | Clearing halos were observed | (Stern & Howard, 2000) |
| Polyurathenase B (PueB) | *Pseudomonas chlorapis* | AF 188366/ Q9R9H2 | Impranil DLN | Rhodamine B agar plates containing 3 g/L of Impranil DLN | Fluorescent halos were observed | (Howard et al., 2001) |
| LC cutinase | Metagenome | HQ704839/G9BY57 | Polyester Elastollan B85A-10 and Elastollan C85A-10 | PU cubes incubated with purified enzyme at 70°C for 200 h | Weight losses of 4.9% and 4.1% for Elastollan B85A-10 and C85A-10 respectively (FTIR) | (Schmidt et al., 2017) |
| Lipase | *Cryptococcus* MTCC 5455 | AB671329/ Q874E9 | Polyester PU (based on poly(diethylene glycol adipate) | Concentrated lipase (1500 U) 96h at 30 °C | 96% weight loss with the production of diethylene | (Thirunavukarasu et al., 2015) |

| | | |
|---|---|---|
| | and 2,4 TDI) | glycol (DEG) |

**Table S5.** Enzymes involved in polylactic acid (PLA) degradation. Reaction conditions and effect on specific materials are reported for each enzyme.

| Enzyme | Origin | GenBank/ PDB ID | Material | Experimental Conditions | Results | Ref |
|---|---|---|---|---|---|---|
| ABO2449 Esterase | *Alcanivorax borkumensis* | YP_694169 | Solid PLA (Sigma-Aldrich) Mw 1.0-1.8 × $10^4$ | 35 °C, 1.0 ml 0.4 M Tris-HCl pH 8.0, 0.1% Plysurf A210G (detergent), 36 h 10-12 mg PLA powder / mL reaction 0.005 g enzyme/g PLA | Up to 90% conversion of PLA into lactic acid monomers and oligomers | (Hajighasemi et al., 2016) |
| Proteinase K | - | Sigma Aldrich | Poly (L-lactide) PLLA electrospun fiber Mn 2.8 × $10^5$ g*mol$^{-1}$ 2 cm×2 cm, 0.27 mm thick | 37 °C, 250 mL 0.05M Tris-HCl pH=8.6, SDS 5 wt.% with respect to PLLA, 9 h Proteinase K 2 μg/mL | 80% weight loss | (Zeng et al., 2004) |
| Alcalase | Porcine liver | Sigma Aldrich (EC 3.1.1.1) d= 1.25 g/mL | PLA fiber (Toray Industries Inc) 0.126 mm thick 30 g/$m^2$ weight | 40°C, 25 mL Tris pH 8.0 and 10 mM CaCl, 21 days 0.02 g PLA/ ml reaction 0.45 g enzyme/ g PLA | 1.4% weight loss | (Lee et al., 2014) |
| Cutinase (PaE) | *Pseudozyma antarctica* JCM 10317 | - | Poly(DL-lactic acid) pellets (Wako Chemicals) Mw 1.3×$10^5$ | 30 °C, 50μl 20 mM Tris– HCl, pH 8.8, 24 h 0.004 mg emulsified PLA/ mL reaction 0.2 mg purified enzyme/ g emulsified PLA | 50.4% mineralization | (Shinozaki et al., 2013) |
| Lipase | *Candida cylindracea* | EC 3.1.1.3 | PLA fiber (Toray Industries Inc) 0.126 mm thick | 40°C, 25 mL Tris pH 8.0 and 10 mM CaCl, 21 days 0.02 g PLA/ mL reaction | Below 5% weight loss | (Lee et al., 2014) |

| | |
|---|---|
| 30 g/m$^2$ weight | 0.45 g enzyme/ g PLA |

**Table S6.** Enzymes involved in polyhydroxybutyrate (PHB)/PHB-copolymers degradation. Reaction conditions and effect on specific materials are reported for each enzyme.

| Enzyme | Origin | GenBank/<br>PDB ID | Material | Reaction conditions | Weight loss (%) | Ref. |
|---|---|---|---|---|---|---|
| PHB depolymerase | *Alcaligenes faecalis* | AAB40611.1 | P(3-HB) from *Cupriavidus necator* (*Alcaligenes eutrophus*)<br>$M_n$ 281000<br>Chloroform-casted film, aged for 3 weeks, $T_m$ 177 °C, $X_c$ 62%<br>10x10x0.07 mm | 37 °C, 1 mL 0.1 M K-Phosphate<br>pH 7.4, 19 h<br>(8 mg film/mL reaction)<br>2 pmol enzyme/mg film or<br>8 pmol enzyme/cm$^2$ film | 22.5 | (Abe & Doi, 1999) |
| | | | 74% isotactic P[(R,S)HB],<br>$M_n$ 21600<br>Chloroform-casted film, aged for 3 weeks, $T_m$ 110 °C, $X_c$ 18%<br>10x10x 0.07mm | | 55 | |
| | | | 63% isotactic P[(R,S)HB]<br>$M_n$ 21600<br>Chloroform-casted film, aged for 3 weeks, $T_m$ 64 °C, $X_c$ 12%<br>10x10x0.07 mm | | 42.5 | |
| | | | P(3-HB) from *Cupriavidus necator*, $M_n$ 228000<br>Chloroform-casted film, aged for 3 weeks, $X_c$ 60%<br>10x10x0.07 mm | 37 °C, 1 mL 0.1 M K-Phosphate<br>pH 7.4, 5 h<br>(8 mg film/mL reaction)<br>2 pmol enzyme/mg film or<br>8 pmol enzyme/cm$^2$ film | 21.3 | (Kasuya et al., 1996) |
| | | | P(4-HB) from *Delftia* (*Comamonas*) *acidovorans*<br>$M_n$ 203000<br>Chloroform-casted film, aged for 3 weeks, $X_c$ 38%<br>10x10x0.07 mm | | 10 | |

| | | | | | | |
|---|---|---|---|---|---|---|
| | | | P(3-HP), $M_n$ 143000<br><br>Chloroform-casted film, aged for 3 weeks, $X_c$ 40%<br><br>10x10x0.07 mm | | 26.3 | |
| | | | P(3-HB) from *Cupriavidus necator* (*Alcaligenes eutrophus*)<br><br>$M_n$ 281000<br><br>Chloroform-casted film,aged for 3 weeks, $T_m$ 176 °C, $X_c$ 62%<br><br>10x10x0.1 mm | 37 °C, 1 mL 0.1 M K-Phosphate<br><br>pH 7.4, 5 h<br><br>(14 mg film/mL reaction)<br><br>1.1 pmol enzyme/mg film or<br><br>8 pmol enzyme/cm$^2$ film | 5.7 | (Abe et al., 1998) |
| | | | P(HB-*co*-5mol%HV), $M_n$ 91000<br><br>Chloroform-casted film, aged for 3 weeks, $T_m$ 147 °C, $X_c$ 50%<br><br>10x10x0.1 mm | | 60.7 | |
| | | | P(HB-*co*-18mol% lactide), $M_n$ 52000<br><br>Chloroform-casted film, aged for 3 weeks, $T_m$ 124 °C, $X_c$ 35%<br><br>10x10x0.1 mm | | 13.6 | |
| PHB depolymerase | *Paucimonas* (*Pseudomonas*) *lemoignei* | 5MLY | P(HB-*co*-10mol%HV)<br><br>Bacterial origin (Marlborough Biopolymers Ltd, England)<br><br>$M_n$ 96000<br><br>Chloroform-casted film, $T_m$ 145 °C, $X_c$ 61%, 25x8x(0.2-0.3) mm | 37 °C, 1.5 mL 50 mM Tris-HCl pH 8, 1 mM $CaCl_2$, 4x20 h<br><br>25 pmol enzyme/cm$^2$ film/20 h | 85 | (Scandola et al., 1997) |
| Lipase | Porcine pancreas (Wako chemical Co., Japan) | - | P(3HB-*co*-98mol%4HB)<br><br>Chloroform cast films, then hot-pressed aged for >2 weeks<br><br>5x5x0.05 mm$^3$ | 37 °C, 3 mL D-PBS pH 7.4, 120 h<br><br>(1-2 mg film/mL reaction)<br><br>2.5 pmol enzyme/mg film or 10 pmol enzyme/cm$^2$ film | 80 | (Chuah et al., 2013) |

**Table S7.** Enzymes involved in poly(butylene succinate) (PBS) degradation. Reaction conditions and effect on specific materials are reported for each enzyme.

| Enzyme | Origin | Source | Material | Experimental Conditions | Weight loss (%) | Reference |
|---|---|---|---|---|---|---|
| Lipase | *Pseudomonas cepacia* | Amano Pharmaceu-tical Co. | PBSL films (97/3 mol%)<br>Mn 4.9 × $10^4$<br>5mm x 60 mm<br>0.2 mm thick | 50 °C, 7.6 ml 0.1 M $KH_2PO_4$/KOH pH 6.0, 14 days<br>0.66 g PBSL / mL reaction<br>1.88 mg enzyme /g PBSL<br>0.016 mg enzyme / $mm^2$ PBSL<br>Or<br>0.471 nmol enzyme / $mm^2$ PBSL | 90 | (Taniguchi et al., 2002) |
| Lipase | *Pseudomonas* sp. | Amano Pharmaceutical Co. | PBSA films<br>Mn 6.4 × $10^4$<br>1 mm× 1mm piece<br>0.1 mm thick | 37 °C, 50mM phosphate buffer pH 7.0, 10 days<br>7.5 U enzyme /$mm^2$ PBSA | 96 | (Tsutsumi et al., 2003) |
| Purified lipase | *Cryptococcus* sp. | - | PBS films<br>Mn 6.0 × $10^4$<br>200 μm thick | 30 °C,10 ml 0.1M phosphate buffer, pH 7.0, 72 h<br>2.6 mg PBS / mL reaction<br>0.63 mg protein / mg PBS | 100 | (Thirunavukarasu et al., 2016) |
| Lipase | *Candida antarctica* | Beijing Cliscent Technology Co | PBS films<br>Mn 10.0 × $10^4$<br>30 mm×10 mm piece<br>0.5 mm thick | 45 °C,10 ml phosphate buffer, pH 7.0, 26 h<br>0.3 U/$mm^2$ PBS | 90 | (Shi et al., 2019) |
| Lipase | *Pseudomonas* sp. | Commercial<br>Lipase PS$^R$ | PBST (90/10 mol %) films<br>Mn 30.0 × $10^4$<br>30 mm×10 mm piece<br>0.1 mm thick | 37 °C, 3 ml 1 mM phosphate buffer, pH 7.0, 15 days<br>0.033 mg enzyme/ mL reaction<br>or | 95 | (Li et al., 2007) |

| | | | | | | |
|---|---|---|---|---|---|---|
| | | | | 0.1 µg enzyme/ mm$^2$ PBST | | |
| Cholesterol esterase | *Pseudomonos fluorescens* | Sigma Aldrich | PBSA films (40/60 mol %)<br>Mn 3.4 × $10^4$<br>6 mm×6 mm piece<br>0.15 mm thick | 37 °C, 1 ml phosphate buffer pH 7.2, 15 days | 15 | (Tserki et al., 2006) |
| Cholesterol esterase | *Pseudomonos fluorescens* | Sigma Aldrich | PBS films<br>Mn 5.95 × $10^4$<br>6 mm×6 mm piece<br>0.15 mm thick | 37 °C, 1 ml phosphate buffer pH 7.2, 15 days | 10 | (Tserki et al., 2006) |
| Cutinase | *Fusarium solani* | - | PBS films<br>Mn 15.0-21.0 × $10^4$<br>30 mm×10 mm<br>0.1 mm thick | 40 °C, 10 mL 20 mM Tris-HCl pH 8.0, 10 h<br>20 µg enzyme/mL reaction<br>0.33 µg enzyme/ mm$^2$ PBS<br>or<br>0.014 nmol enzyme/ mm$^2$ PBS | 100 | (Hu et al., 2016) |
| Cutinase | *Fusarium solani* | - | PBS films<br>Mn 10.0 × $10^4$<br>30 mm×10 mm piece<br>0.5 mm thick | 37°C, 10 ml phosphate buffer, pH 7.0, 26 h<br>0.3 U/mm$^2$ PBS | 100 | (Shi et al., 2019) |

**Table S8.** Enzymes involved in polycaprolactone (PCL) degradation. Reaction conditions and effect on specific materials are reported for each enzyme.

| Enzyme | Origin | Material | Experimental Conditions | Results | Reference |
|---|---|---|---|---|---|
| Lipase | *Pseudomonas* | PCL synthesized by ring-opening polymerization<br>Mn 12.5 × $10^4$<br>Mn 5.7 × $10^4$<br>Mn 2.4 × $10^4$<br>0.1-0.3 mm thick<br>10 mm x10 mm film | 27 °C, 15 mL 0.025 M phosphate buffer pH 7.0, 100 h<br>0.66 mg PCL / mL reaction<br>0.75 g enzyme/g PCL<br>0.0375 mg enzyme/$mm^2$ PCL | 100% weight loss for all samples | (Gan et al., 1997) |
| Lipase | *Mucor miehei* | PCL (Sigma–Aldrich)<br>$M_n$ 87,000 | 40 °C, Toluene 4 mL, $H_2O$ 1.5 mL, 24 h<br>0.07 g PCL/mL reaction<br>0.1 g enzyme/g PCL | 74% $M_n$ reduction | (Pastorino et al., 2004) |
| Cutinase | *Aspergillus fumigatus* | PCL film (mixing dichloromethane (20%) and ω-caprolactone monomer)<br>250 µm thick<br>1 $cm^2$ | 40°C, 2,6 mL Tris pH 8.0, 6 h<br>0.011 g PCL/mL reaction<br>0.012 g enzyme/ g PCL<br>0.002 mg enzyme / $mm^2$ PCL | 100% weight loss | (Ping et al., 2017) |
| Cutinase | *Fusarium solani* | PCL film (mixing dichloromethane (20%) and ω-caprolactone monomer<br>250 µm thick<br>1 $cm^2$ | 40°C, 2.6 mL Tris pH 8.0, 6 h<br>0.011 g PCL/ ml reaction<br>0.012 g enzyme/ g PCL<br>0.002 mg enzyme/$mm^2$ PCL | 44.3% weight loss | (Ping et al., 2017) |
